# Newly discovered crocodile mummies of variable quality from an undisturbed tomb at Qubbat al-Hawā (Aswan, Egypt)

**DOI:** 10.1371/journal.pone.0279137

**Published:** 2023-01-18

**Authors:** Bea De Cupere, Wim Van Neer, Vicente Barba Colmenero, Alejandro Jiménez Serrano

**Affiliations:** 1 Operational Directorate Earth and History of Life, Royal Belgian Institute of Natural Sciences, Brussels, Belgium; 2 Laboratory of Biodiversity and Evolutionary Genomics, University of Leuven, Leuven, Belgium; 3 Departamento de Antropología, Geografía e Historia, University of Jaén, Jaén, Spain; Istanbul University: Istanbul Universitesi, TURKEY

## Abstract

A description is provided of the crocodile remains that were found during an excavation carried out in 2019 at Qubbat al-Hawā (Aswan, Egypt). The material consists of five more or less complete bodies and five heads that were in varying states of preservation and completeness. The absence of resin, which was apparently not used during the preparation of the mummies, and the almost complete loss of linen bandages, due to insect damage, allowed a detailed morphological and osteometric description of the remains. Attention was focused on the general state of preservation of the crocodiles, the completeness of their skeletons and skulls, the presence of cut or other marks that could indicate the cause of death, and the processing of the carcasses. Moreover, the possible provenance of the crocodiles, the methods of capture and killing of the animals and their possible chronological attribution are discussed. It is concluded that the manner in which these specimens were prepared, as well as the variation observed in the type of ‘final product’, are unlike any other crocodile material described so far. The preparation method suggests a pre-Ptolemaic date for the deposit. The morphological and metrical features indicate that both *Crocodylus niloticus* and the recently resurrected species *Crocodylus suchus* are present among these individuals that range from 1.8 to 3.5 m in length.

## Introduction

**Fig 1:**
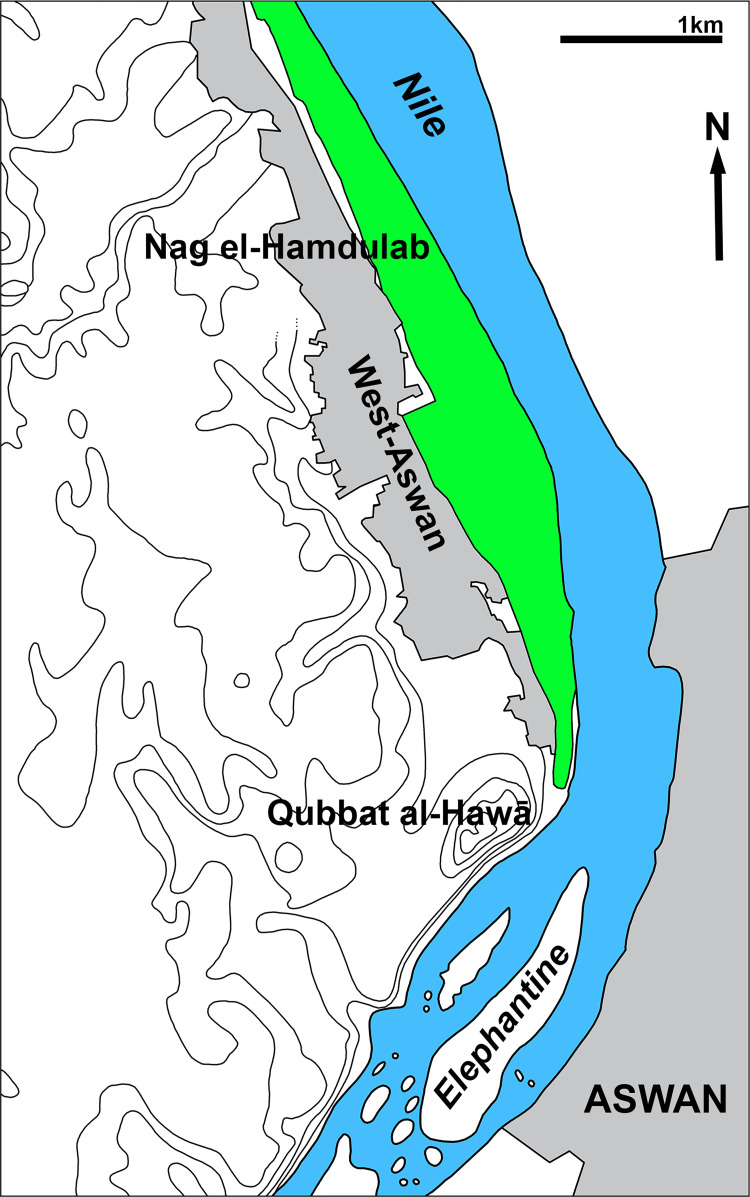
The area of Aswan with indication of Qubbat al-Hawā.

Since 2008, a team of the Universidad de Jaén (Spain) has been conducting fieldwork in the rock tombs of Qubbat al-Hawā, a site on the west bank of the Nile opposite the town of Aswan (Fig 1). The rock cut tombs in which dignitaries of the region were buried, date mainly from the Old and Middle Kingdom, although there is also evidence of occupation of the site during the New Kingdom, the Late Period and Byzantine period [1]. Faunal remains are relatively rare at the site and consist largely of intrusive animals that entered the tomb deposits through natural processes. Cattle skulls and foot extremities predominate among the anthropogenic remains, and in one case a complete left foreleg was found [2]. Such finds of Egyptian longhorn cattle, which may be considered as funerary gifts, were also briefly reported in earlier excavations in the 1960s [3].

In this paper, we report on an exceptional deposit of crocodiles found during the 2019 excavations in an undisturbed tomb. The finds include five more or less complete, large animals as well as five skulls of also relatively large crocodiles that provide unique information, despite the fact that numerous crocodile mummies have been discovered in Egypt for centuries. Kessler [4] and Ikram [5] cite more than 20 animal cemeteries in Egypt where crocodiles were buried, although in some cases there is only literary evidence. Crocodiles were also sporadically found at several sites where the focus was on other species. Burial sites associated with the cult of Sobek, from which abundant crocodiles were reported, are located in the Fayum (Hawara, Umm el-Baraqat (Tebtynis), Al-Lahun, …), and farther south in the es-Samun caves (al-Maabda), in Esna and Kom Ombo [6, 7]. The latter locality is at about 50 km north of Aswan whereas Esna is at about 150 km as the crow flies. Also at Elkab, near Edfu at c. 110 km north of Aswan, a crocodile sanctuary is believed to have existed as shown by both textual and archaeological evidence [8].

Many museums worldwide have Egyptian animal mummies in their collections and among them are often crocodiles [9]. These specimens were gathered in large numbers, especially in the late 19^th^–early 20^th^ century. The Egyptian Museum in Cairo and the Musée des Confluences in Lyon possess the largest collections of animal mummies, albeit that in many cases the exact origin of the specimens is unknown [10, 11]. A general survey of the material at the Musée des Confluences shows that of the 300 or so crocodile mummies, about 80% are from juvenile, often fairly recently hatched specimens. Ten percent are complete, larger specimens (between about 50 and 350 cm total length) and the remaining 10% are heads, skulls or lower jaws [12]. These proportions are an indication of the material stored in the museum that was contributed by Lortet and Gaillard and therefore do not necessarily reflect the proportions in which these animals occurred in cemeteries where they were used as votive offerings. The exact provenance of many of the crocodiles kept in the Musée des Confluences is unknown, but it is likely that most of them come from Esna [13] and Kom Ombo [14].

Although the practice of mummifying crocodiles in Egypt has been reported since Antiquity by historians such as Herodotus, Diodorus, Strabo, Pliny the Elder, Plutarch and Aelian [7], a review of the literature shows that the amount of detailed morphological observations on the crocodiles themselves and on the mummification techniques is quite limited. To avoid unwrapping the mummies, traditional radiographs and CT-scans were performed on crocodiles from the Manchester Museum [15], the British Museum [16] and the Musée des Confluences [17, 18]. It is striking that, unlike many of the other animal species collected by Lortet and Gaillard, almost no osteomorphological or osteometric work has been done on the crocodiles [14]. Indeed, these authors used the animal mummies as a source of information for their study of contemporary Egyptian fauna and therefore the specimens at their disposal were often unwrapped.

In addition to the mummies mentioned above, information on ancient crocodiles can also be obtained from archaeozoological studies. However, crocodiles from more recent excavations of funerary contexts are quite rare and consist only of scattered remains from disturbed contexts. Examples are the Middle Kingdom remains from a necropolis in the valley of North Asasif, Thebes [19], and a rock tomb at Deir el Barsha [20]. Despite the large number of crocodile mummies reported from al-Maabda since the late 19^th^ century, osteological information is limited to an assemblage of heavily burnt remains that allowed only a small number of osteometric or other observations [21]. From Tuna el-Gebel a few isolated cranial and postcranial remains, without any find context, have been reported; the largest specimens were from crocodiles with an estimated total length of 4.5 and 5.5 m [22]. Finally, at Elkab, numerous burnt crocodile bones have been found from animals thrown from a number of rock-cut tombs used as repositories for animal mummies in the Greco-Roman period [23, 24]. The minimum number of individuals was not given, but the reconstructed total lengths were estimated between 1 and more than 5 m.

Given the paucity of information in the literature, it is clear that the in situ finds of Qubbat al-Hawā may provide unique data.

### The site and the excavation of the crocodile deposit

**Fig 2:**
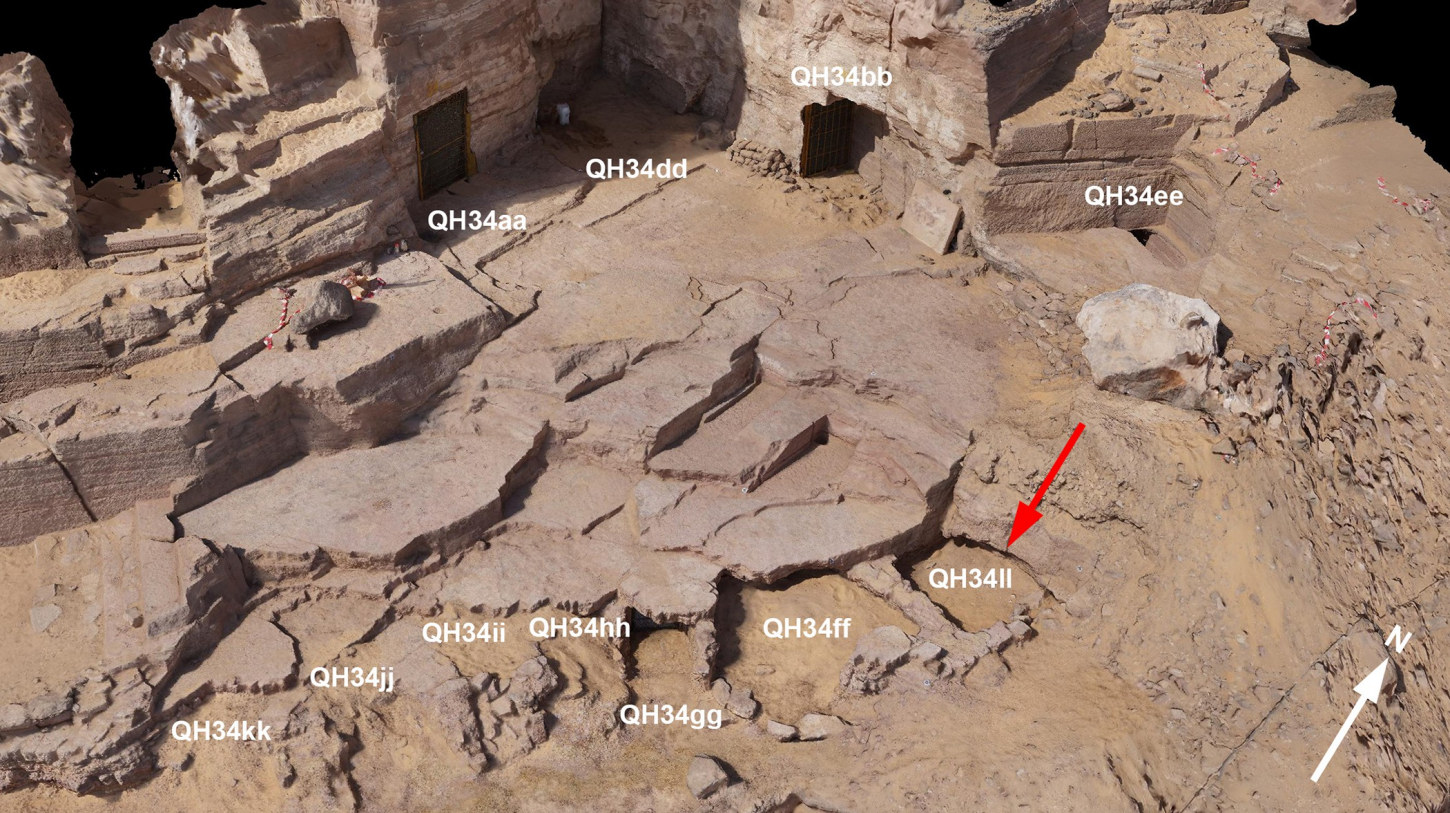
Orthophotography of the tombs located on the hillside, Campaign 2019. The arrow indicates the tomb where the crocodiles were found.

Excavations carried out since 2018 in the eastern area of Qubbat al-Hawā have revealed seven new intact burial chambers discovered under a thick Byzantine rubbish dump. These small rock tombs (QH34ff, QH34gg, QH34hh, QH34ii, QH34jj, QH34kk and QH34ll) were located below the level of the rocky platform that lies in front of two larger Middle Kingdom graves (QH34aa and QH34bb) (Fig 2). The undecorated tombs were cut into the weak layers of lutites at the edge of the hillslope before the 12^th^ Dynasty [25]. The roof of some of them, including Tomb QH34ll, collapsed, making it necessary to excavate from the top. Tomb QH34ll appears to be a small space (2 x 1.80 m), with an entrance on the east side, facing the slope of the hill. A first stratigraphical layer in the tomb (stratum EU 445) yielded the remains of several crocodiles. Some were in good condition and even showed signs of being mummified, as remains of wrappings (bandages and mats) occurred. However, no associated artefacts, such as ceramics, were found that could be used to date the crocodile remains. Below this level with the crocodiles, a sandy layer was discovered (stratum EU 446). It was probably deposited to level the floor of the tomb for the purpose of positioning the crocodiles. A considerable number of elongated faience beads from the New Kingdom were collected from this sand layer. Below this was stratum EU 447, which contained the deposit of four human burials whose imprint of the wooden coffins was still preserved. Associated medium-sized ceramic bowls indicate that these burials date from the end of the First Intermediate Period.

All necessary permits were obtained from the Ministry of Tourism and Antiquities, Arab Republic of Egypt, for the described study, which complied with all relevant regulations. The crocodile finds from tomb QH34ll have been studied on site and are also currently stored there.

## Description of the finds

**Fig 3:**
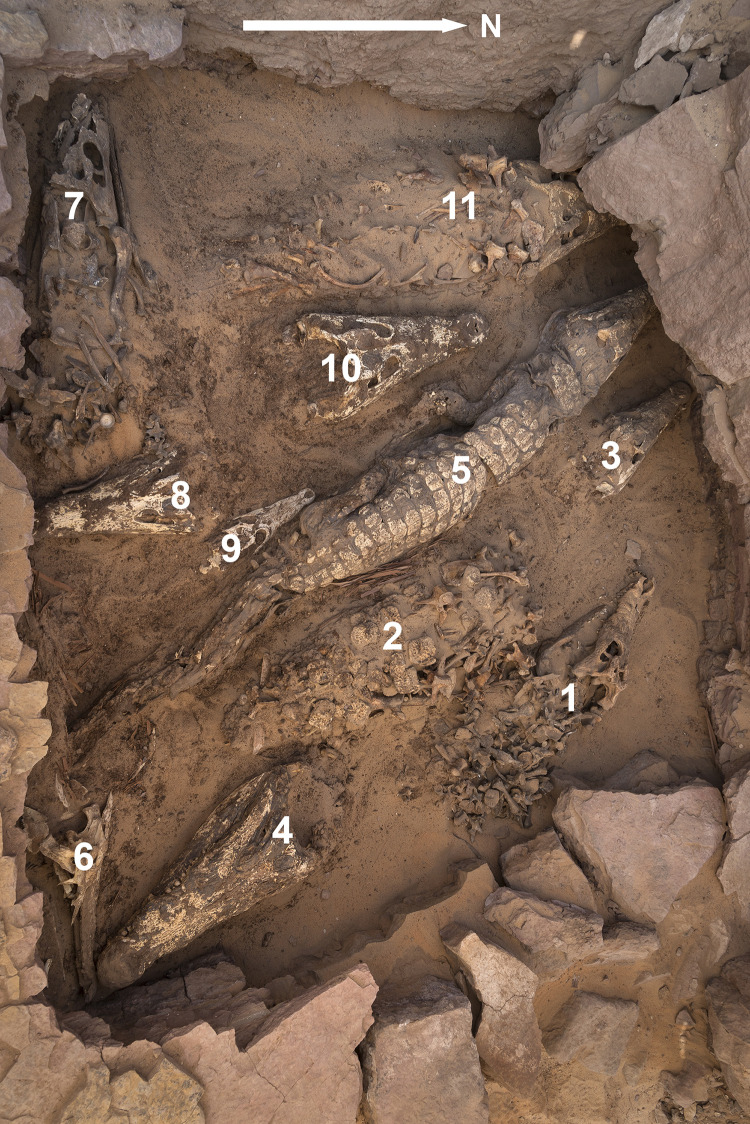
Overview of the crocodiles during excavation showing numbers attributed to them before lifting.

In the course of the excavation, carried out under the supervision of VBC, numbers were assigned to what were believed to be the remains of different individuals (Fig 3). When the material was analysed by BDC and WVN in spring 2022, the numbers assigned to the different clusters were maintained. The original numbering of the crocodiles has also been maintained in the description below, albeit that instead of 11 individuals there appear to be only the remains of 10 crocodiles. These were five isolated skulls, as well as four more or less complete skeletons and a single, still articulated, mummified specimen. Crocodile #2 has no head and the skull was found to be labelled as “crocodile #4”. In the further description below and in the supplementary information, all bones are assigned to crocodile #4, and crocodile #2 is not maintained as a separate individual.

**Fig 4:**
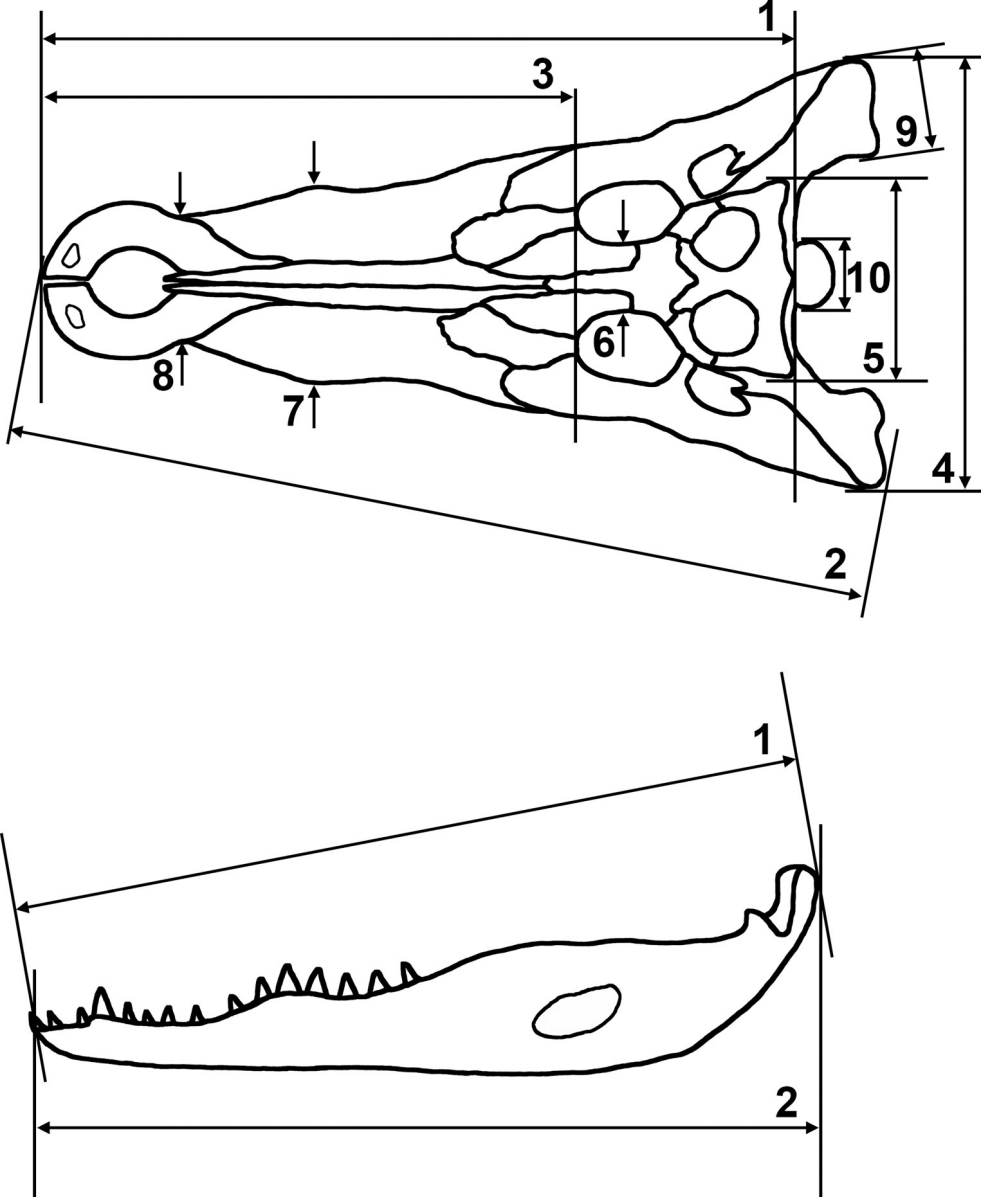
Measuring distances defined for the skull and lower jaw. **Skull:** 1) head length (anterior border premaxilla to posterior margin supraoccipital); 2) greatest lateral length of skull (anterior margin of premaxilla to posterior margin of quadrate); 3) snout length (oral border of orbita to anterior margin of premaxilla); 4) greatest posterior breadth over quadratojugalia; 5) greatest posterior breadth over squamosa; 6) smallest breadth of the frontals; 7) greatest breadth of snout (greatest anterior breadth over the maxillae); 8) smallest breadth of snout (smallest breadth on junction of maxilla and premaxilla); 9) greatest breadth of quadratum; 10) greatest breadth of occipital condylus. **Lower jaw**: 1) greatest length of lower jaw (anterior margin of dentary to posterior margin of retroarticular process); 2) greatest length of lower jaw (idem in measuring box); 3) greatest breadth of articular (not shown on figure).

The following is a description of the crocodile remains discovered. Attention is paid to the general preservation of the material, the skeletal elements represented, pathological deformations, and cut marks. Measurements were taken whenever possible. The skull and lower jaw measurements, defined in Fig 4, are given in Table 1. The postcranial measurements are listed in S1 Table. These include, for the long bones, the greatest length (GL), the greatest proximal width (Bp) and the greatest distal width (Bd). The measuring distances defined for the elements of the pectoral and pelvic girdle, the hyoid, the first cervical vertebrae, the astragalus and calcaneus are shown in S1 Fig. Most of the measurements were taken with callipers and had a precision of 1 mm or 0.1 mm, while the longer cranial distances were taken with a metal tape measure with a precision of 5 mm. A schematic representation of a crocodile skeleton with indication of the main elements is shown in S2 Fig.

**Table 1:** Overview of the measurements in mm taken on the skulls and lower jaws.

**measurement**										
**skull**	#11	#4	**#10**	#1	**#3**	**#6**	**#8**	#5	#7	**#9**
1	505	500	475	405	405	390	385	375	-	264
2	555	580	540	450	460	440	420	420	-	287
3	345	350	330	285	277	275	260	250	-	177
4	298	288	263	218	209	206	195	215	-	127
5	136	130	123	103	-	102	92.5	95	-	67
6	49.5	42	47	38	37	-	32	37	-	19
7	155	140	131	99	101	103	96	106	-	59
8	96	87	85	55	62	60	58	64	-	35
9	61	(57)	53.5	45.5	41	41	38.5	43	37	26
10	36	35	34.9	32	29.5	28	-	-	27	20
**lower jaw**										
1	630	635	605	500	495	-	465	460	410	315
2	620	620	585	485	490	-	450	445	395	310
3	66	70	55	47	57	-	41.5	-	38.5	26

Measurements are arranged from the largest (at the left) to the smallest individual. Measuring distances are shown in Fig 4. Measurements in brackets are approximate. The skulls in bold are from crocodiles that were only represented by their heads.

An estimate of the total length of the crocodiles is given, based on the ratio of head to total length. According to Hutton [26] the 1:7 ratio of skull length to total length observed in Zimbabwe also applies to other populations. A study on *Crocodylus porosus* shows that the ratio increases to some extent as crocodiles grow and can reach 1:8 for animals above 5 m [27], but overall the ratio observed in Zimbabwe can be safely maintained. Estimates of length are useful because they give an idea of the size of crocodiles that people could hunt and make into mummies. Moreover, the reconstructed lengths are important when discussing the species identifications below.

### The mummified complete individual

**Fig 5:**
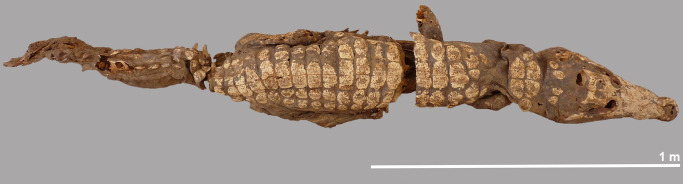
Dorsal view of the complete crocodile #5.

**Fig 6:**
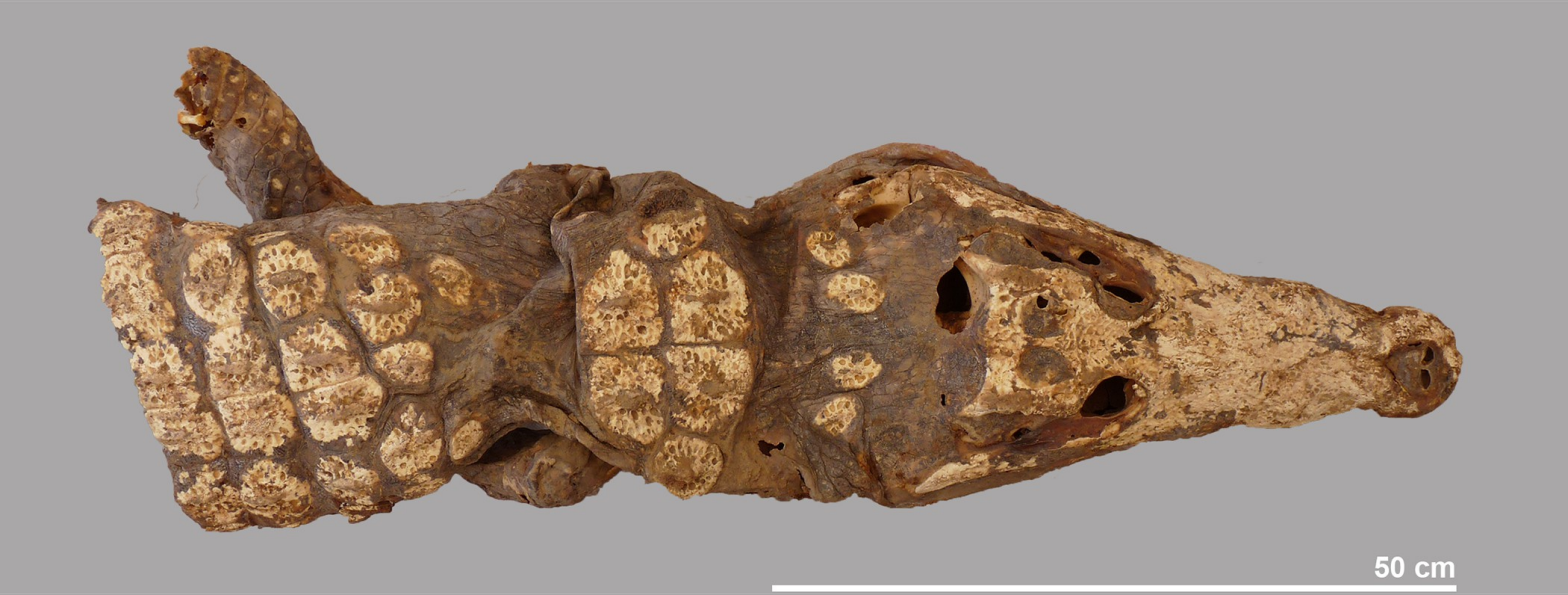
Dorsal view of the head and anterior part of crocodile #5.

**Fig 7:**
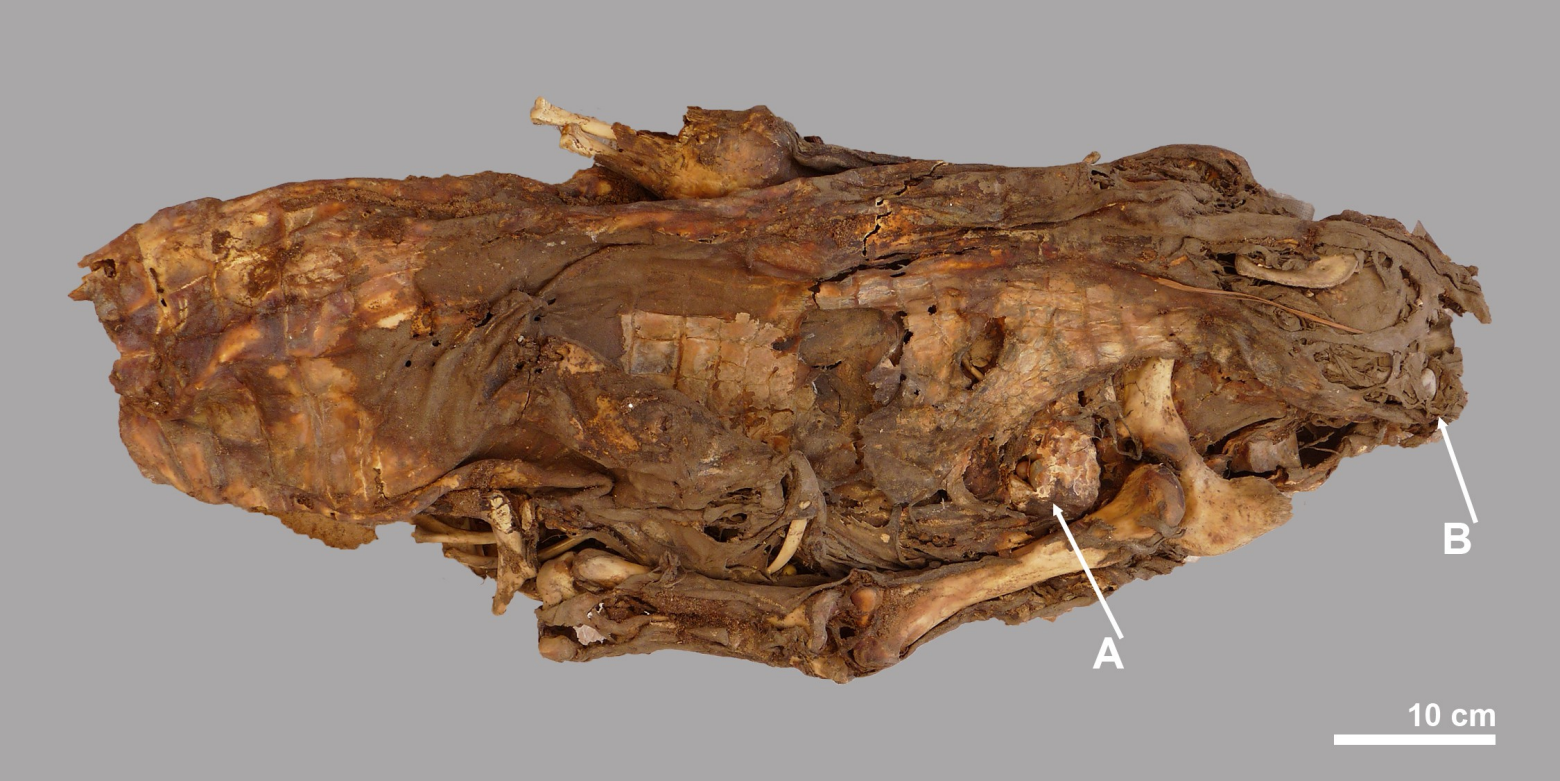
Ventral view of the mid-part of crocodile #5. The bones of the right hind leg and the articulating ischium are visible. The left hind leg is still almost completely covered with skin. The skin on the ventral side of the body is largely intact and shows some folds. Gastroliths could be observed in the place of the arrows (see Fig 8).

**Fig 8:**
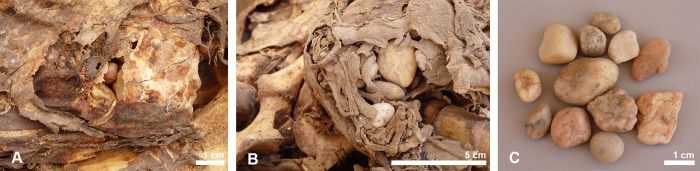
Area A (left) and B (centre) of the ventral part of the body of crocodile #5 with gastroliths visible in situ. Three gastroliths are visible in area A, but more are visible on the right, protruding through the soft tissue. Several gastroliths from area B have fallen out of the body and are shown separately (C, right).

The best preserved specimen, labelled crocodile #5, was still largely articulated in the deposit and could be lifted in its entirety, although the individual had a tendency to fall apart into three main pieces (Figs 3 and 5). Taphonomic damage to the skin was almost negligible. The head, the neck region and the anterior part of the body–up to the level of the fourth row of dorsal scutes–form one unit (Fig 6). The four post-occipital osteoderms are in a semi-circle, and there are six nuchals in two rows. Both forelegs are still present, but the extremities are damaged and part of the phalanges are missing. The right foreleg is folded backwards along the body, while the left foreleg is turned laterally. The mid-part of the body, with 13 rows of scutes, has the hind legs folded forward under it. Again, the distal parts of the feet are incomplete. The skin on the ventral side is largely intact with not a single trace of human modification (Fig 7). Finally, the third piece is the tail whose end is missing. Using a tape measure we estimated the minimum total length of the crocodile to be 2.23 m, which is obviously less than the total length of 2.63 m reconstructed from the head length. An interesting observation with this specimen is the presence of gastroliths in situ which can be seen in two instances on the ventral side (Fig 8). Apart from the skull, few skeletal elements could be measured due to skin adhering to the bones.

### Four skeletons

#### Crocodile #4

**Fig 9:**
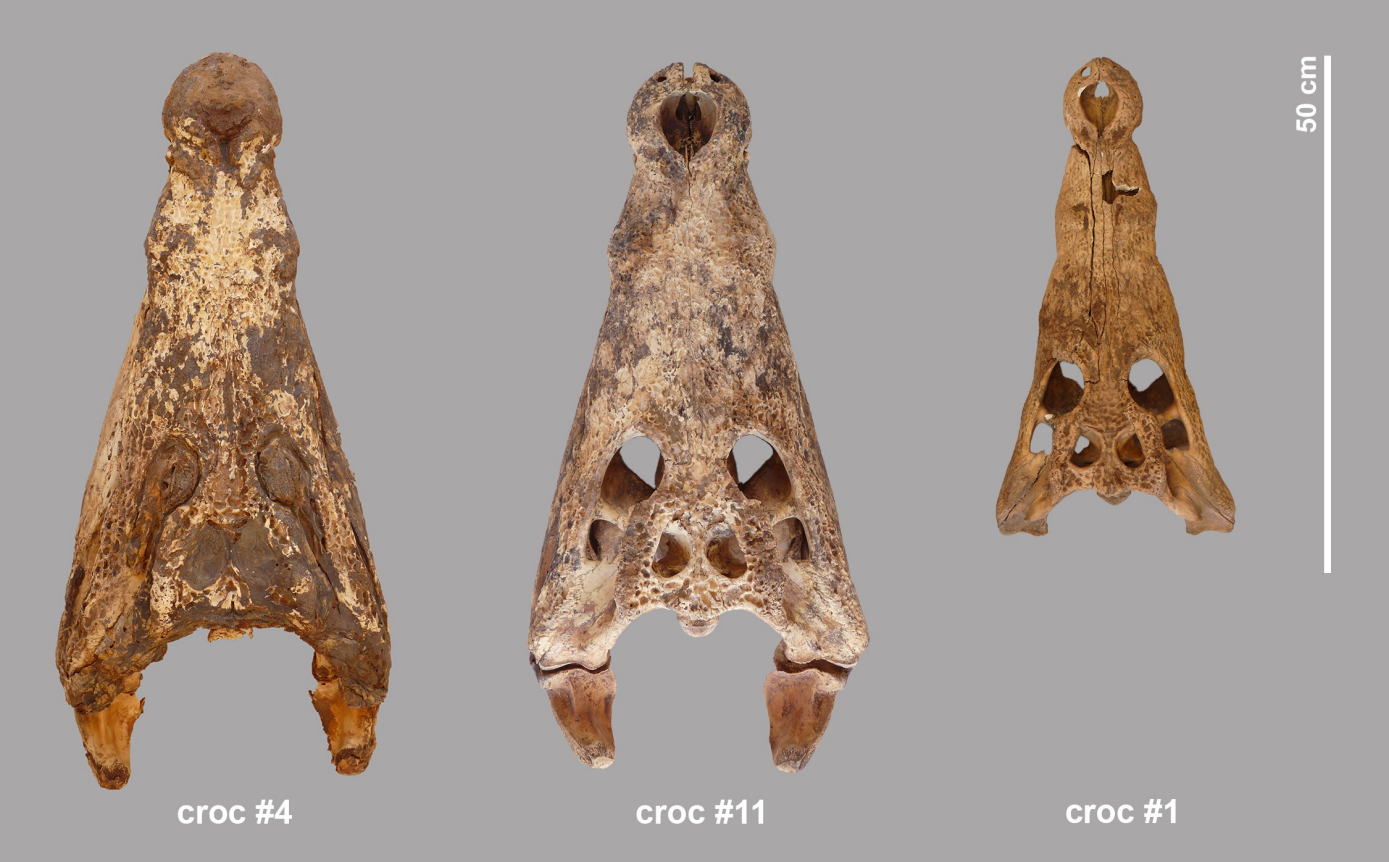
Dorsal view of the skulls of crocodiles #4, #11, and #1.

**Fig 10:**
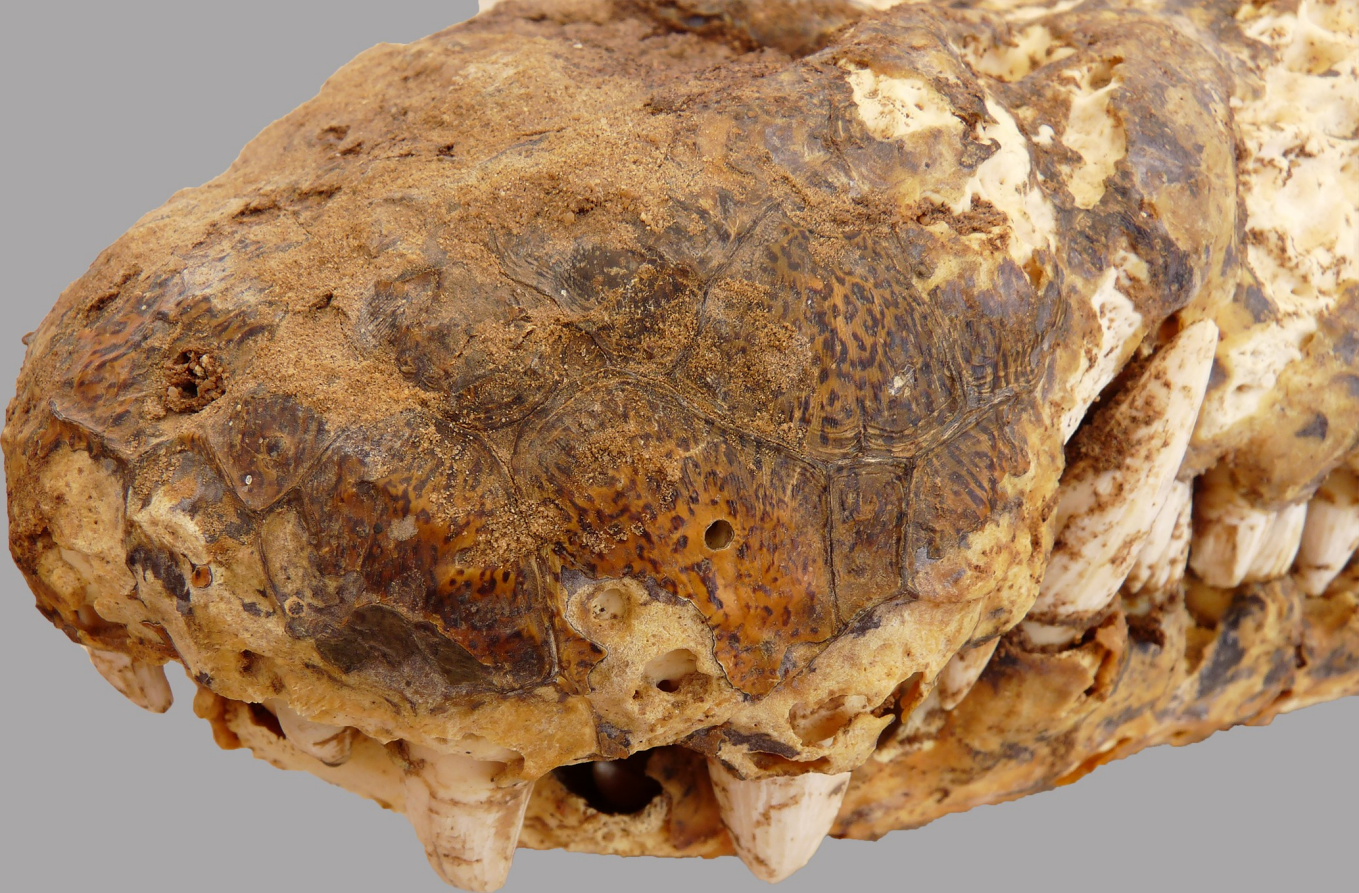
Detail of the left anterior part of the snout of crocodile #4 showing the excellent preservation of the scales, including the colour.

**Fig 11:**
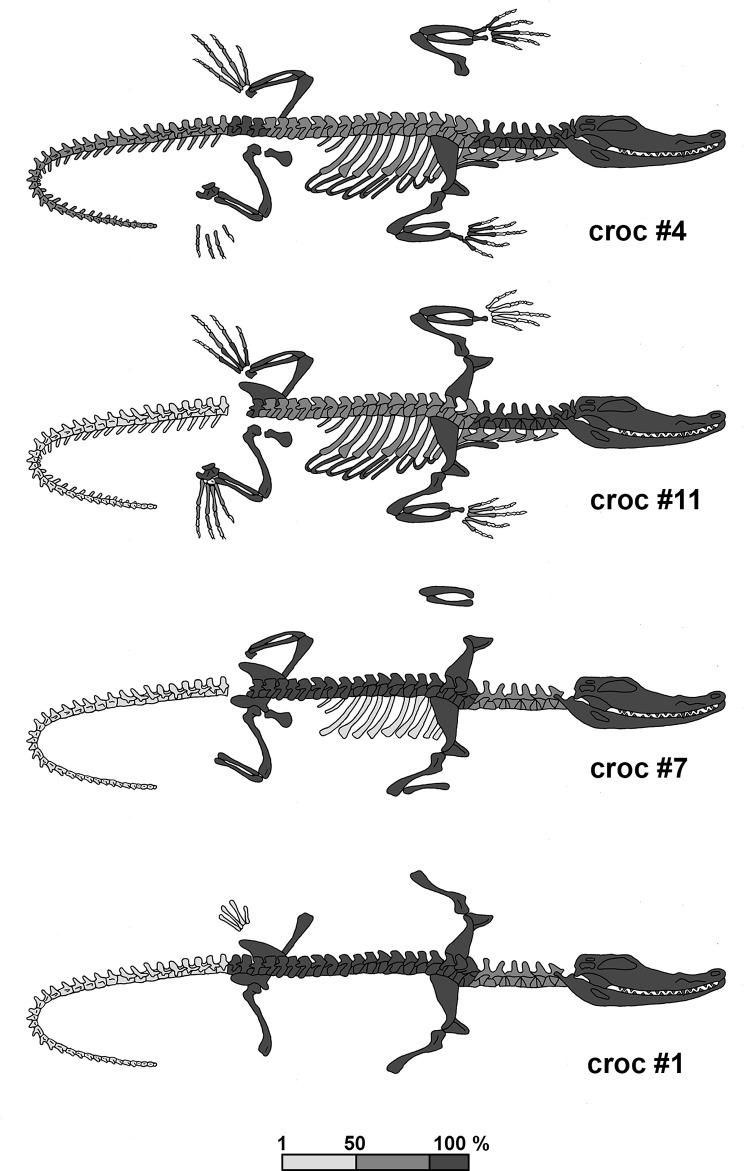
Schematic representation of the skeletal completeness of crocodiles #4, #11, #7 and #1. Missing elements are not shown and for the others the degree of completeness is given as a percentage of the expected numbers, see S2 Table.

The most complete skeleton is from crocodile #4, an individual with an estimated length of 3.50 m based on its skull dimensions. The skull, including the lower jaws, is well preserved with soft tissue still visible at the level of the nostrils, the eye sockets, and the lateral temporal and supratemporal fenestrae (Fig 9). In certain areas, such as the snout and the lateral side of the lower jaws, the colour of the scales is still preserved (Fig 10). The proatlas was attached to the occipital condyle. The atlas, axis and all the other cervical vertebrae were also found. The rest of the vertebral column is present with the exception of that from the tail end: 23 of the 37 expected caudal vertebrae were counted. Except for the right hindfoot and the right scapula, all skeletal elements of the pectoral and pelvic girdles and of the fore- and hindlegs were present (Fig 11). A total of 116 dermal scutes were counted. Besides the bones, a piece of soft tissue was also found, consisting of skin attached to the phalanges of two digits.

**Fig 12:**
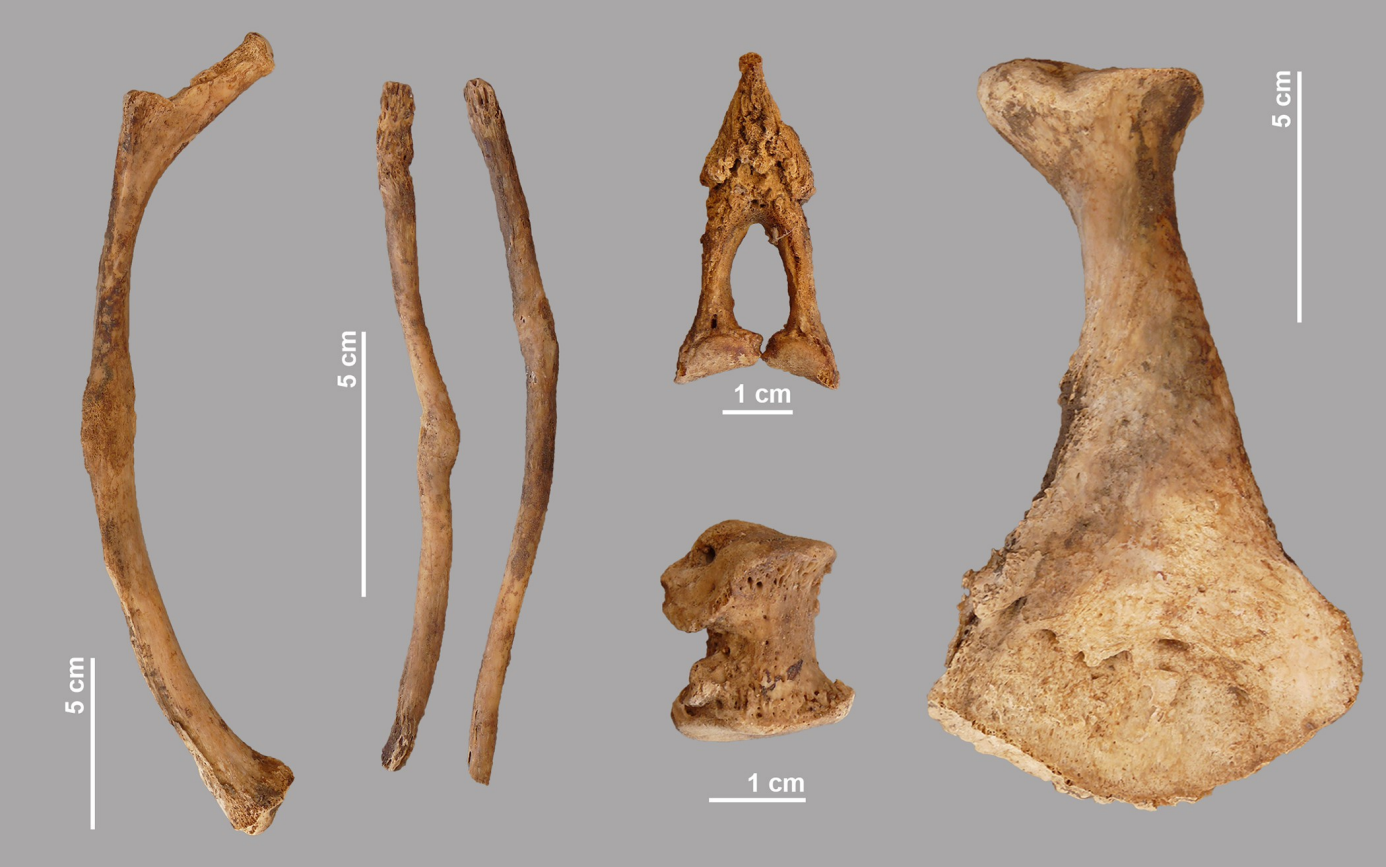
A selection of pathologies observed in crocodile #4. A left dorsal rib and two ventral ribs with a healed fracture; lesions in a left ulnare; new bone formation in a chevron and on the external side of the distal left pubis.

The skeletal elements of this crocodile show several pathologies. Two of the 22 dorsal ribs present show deformities: one left rib has a healed fracture and another left specimen shows an abnormal superficial thickening (Fig 12). Of the 19 ventral ribs present, two specimens have a healed fracture. The distal part of the left pubic bone is covered with additional bone formation. Lesions are also seen on the left ulnare, on the distal part of a left metacarpal III and on a second or third posterior phalanx. Four of the five chevrons found have a pathology: in one specimen the bone is clearly broken and healed, while superficial new bone formation is observed in three others.

#### Crocodile #11

The skull and lower jaws of this individual are intact, with almost no skin preserved (Fig 9). The skull length gives an estimated total length of 3.53 m, which is the largest animal found in the assemblage. Almost all dorsal and cervical ribs are present as well as numerous ventral ribs. All vertebrae are found, except the proatlas and the caudal vertebrae of which less than half are present. Few elements of the foot extremities are missing. The number of dermal scutes (n = 59) is lower than that of crocodile #4. However, it is worth mentioning that the final cleaning of the excavated surface revealed another 83 large scutes that may have belonged partly to crocodile #11. Like crocodile #4, the skeleton of crocodile #11 is quite complete, with even smaller elements such as phalanges present to some extent (Fig 11). This crocodile seems to have been deposited in its entirety, with the exception of the end of its tail.

**Fig 13:**
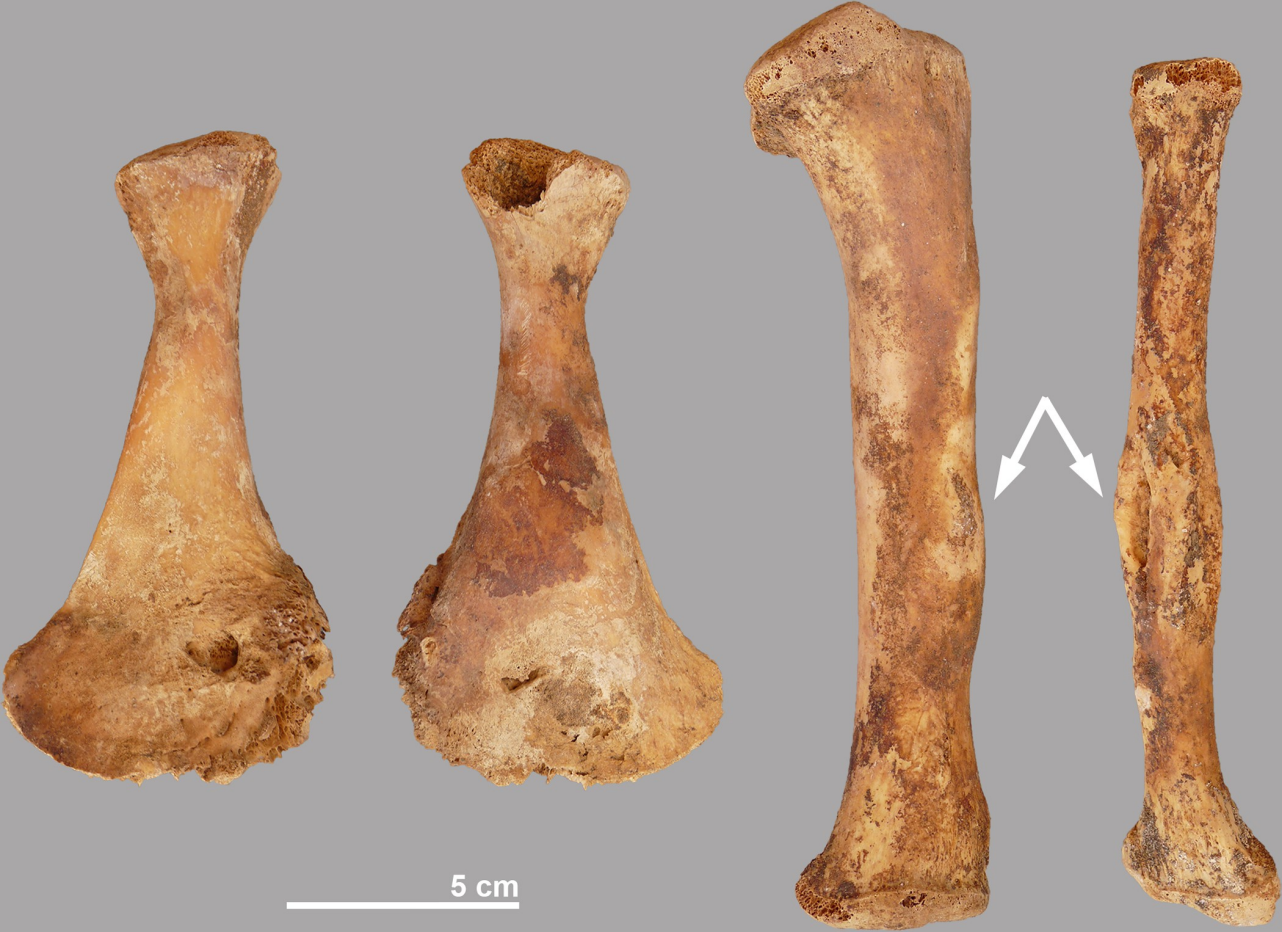
Some of the pathologies seen in crocodile #11. Internal and external side of left pubis with bone infection, possibly osteomyelitis; left tibia with swollen shaft and left fibula with healed fracture.

Crocodile #11 shows pathological deformations to some of its bones. The left pubis has a bony inflammation on both the internal and external side reminiscent of osteomyelitis (Fig 13). A slight exostosis can be seen in the distal part of the right femur. The left tibia and fibula show a lesion that may be related to the same traumatic event: the fibula has a healed fracture in the mid-part of the shaft and a swelling is seen at the same level of the tibia, which appears to be due to a haematoma. Bone lesions are also observed in the distal part of the left metatarsal II and in the proximal part of the right metacarpal III.

#### Crocodile #1

Based on the skull length, the total length of crocodile #1 can be estimated at 2.83 m. This specimen consists of bone only, no remains of skin or other soft tissue are preserved (Fig 9). The lower jaws are detached from the skull. The skeleton of this animal is far from complete (Fig 11). The vertebral column is fairly well represented, only the anterior cervical vertebrae and the last caudals are missing. Twelve of the expected 37 caudal vertebrae were found, the posterior-most elements are absent. Remarkably, no ribs or chevrons at all are found. The skeletal element representation of the fore- and hindlimbs is also peculiar. Both humeri and femurs are present, but the left and right radius and ulna are missing, as are both tibiae and fibulae. Foot elements are almost entirely missing: only two metatarsals were found and metacarpals and phalanges are entirely missing. However, there is a considerable number of dermal scutes (n = 75). No pathologies were observed in crocodile #1.

#### Crocodile #7

**Fig 14:**
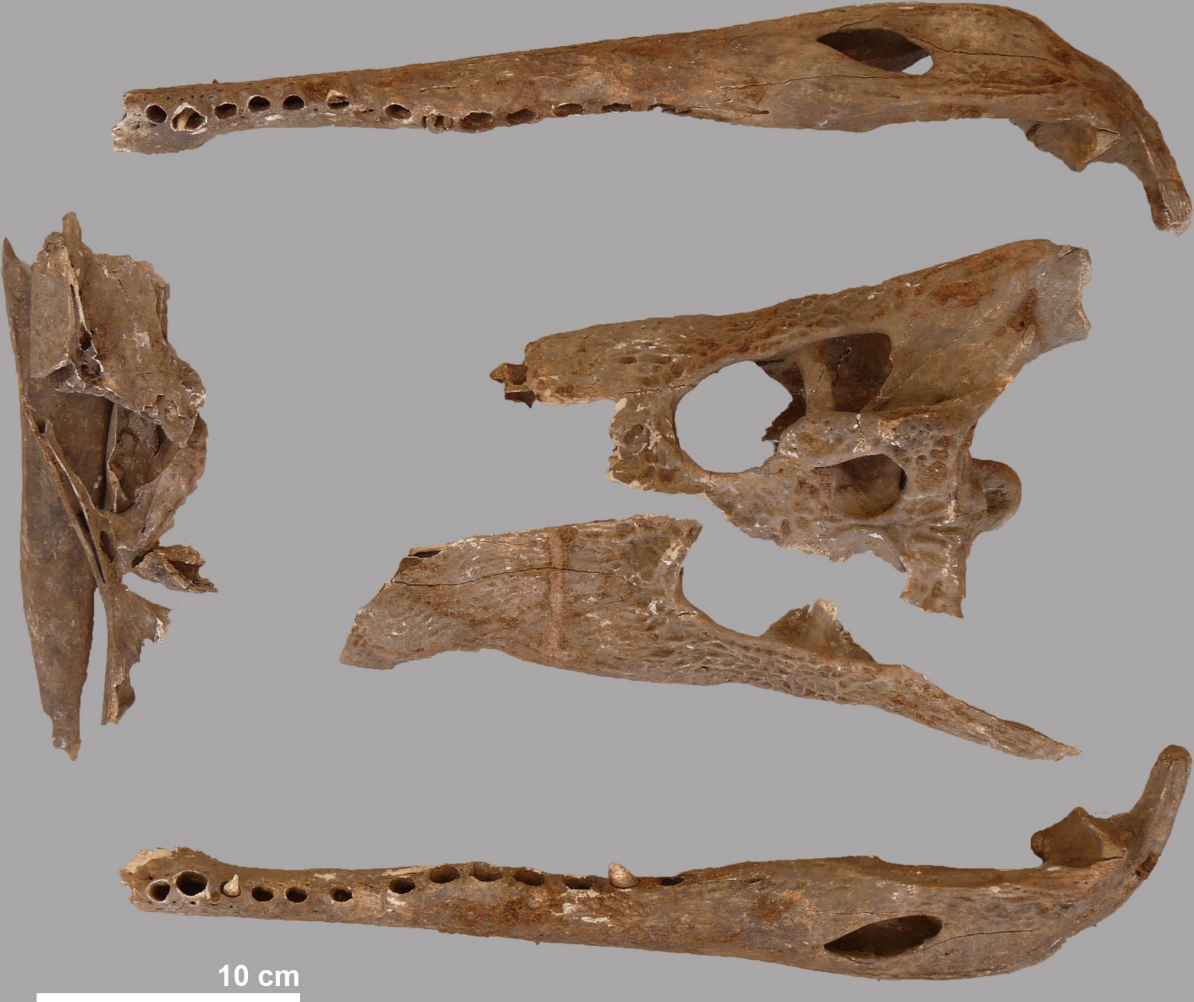
Fragmented skull and lower jaws of crocodile #7.

Because the skull of this crocodile is broken and incomplete (Fig 14), the total length could not be estimated on the basis of the conversion factor defined by Hutton [26]. However, the lower jaws are completely preserved and using the proportion of the head length and lower jaw length seen in the other specimens, the total length of the crocodile must have been around 2.3 m. No teeth are present in what is preserved of the left premaxilla and maxilla, in both the left and right lower jaw only two teeth can be seen. The postcranial skeleton of this crocodile is far from complete (Fig 11). The mid portion of the vertebral column is present, but the first five cervicals, one of the sacral vertebrae and 31 of the 37 expected terminal caudal vertebrae are lacking. Ventral ribs are totally absent and only five left and five right dorsal ribs are recorded. Bones of the pectoral and pelvic girdles as well as from the fore- and hindlegs are found, but many elements are missing. Except for a left astragalus and a right metatarsal I, nothing of the fore- or hindfeet is found. Also the scutes (n = 9) are heavily underrepresented. No pathologies were observed on this specimen.

### Five isolated skulls

#### Crocodile #10

**Fig 15:**
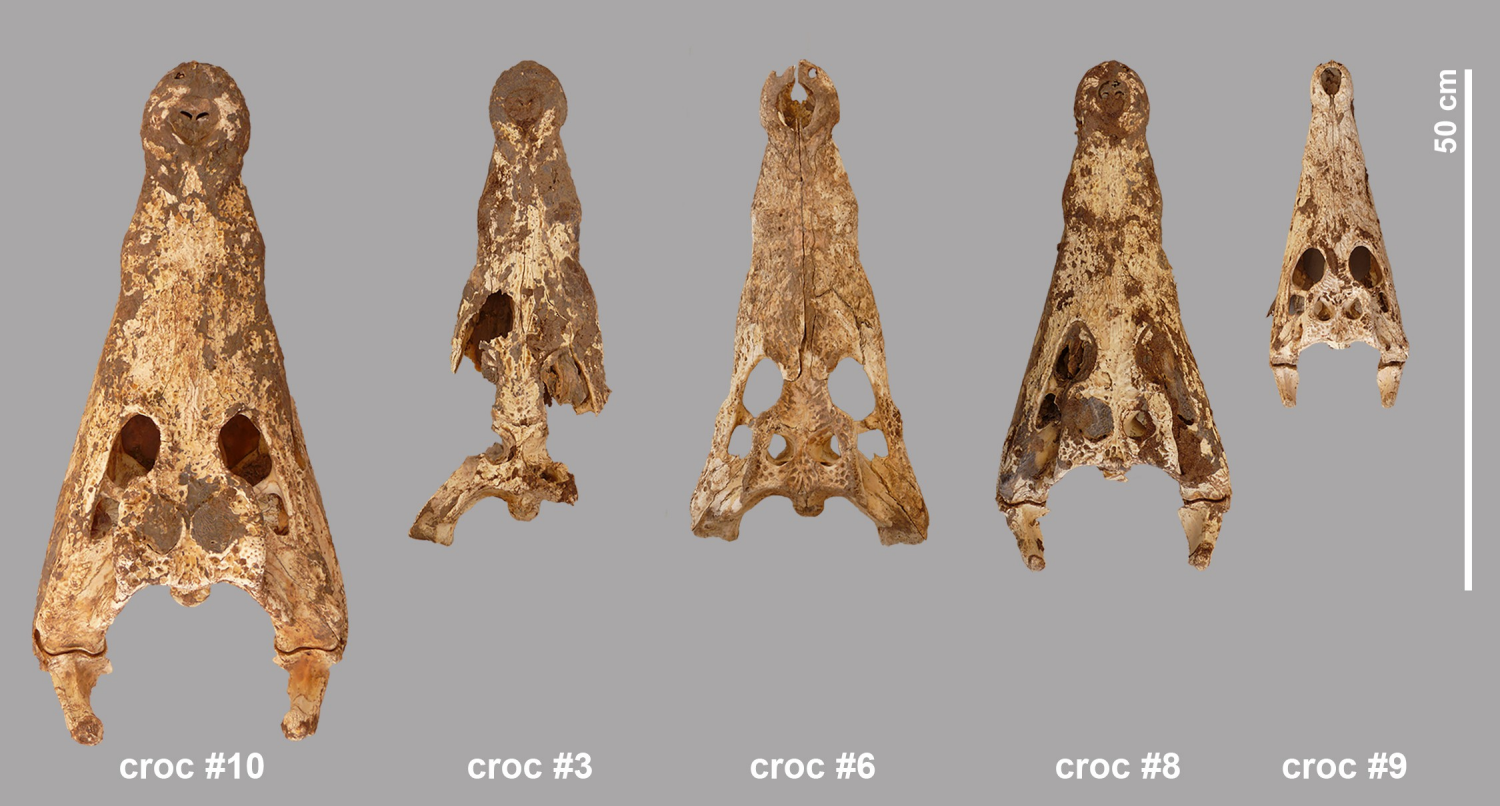
Dorsal view of the skulls of crocodiles #10, #3, #6, #8 and #9.

**Fig 16:**
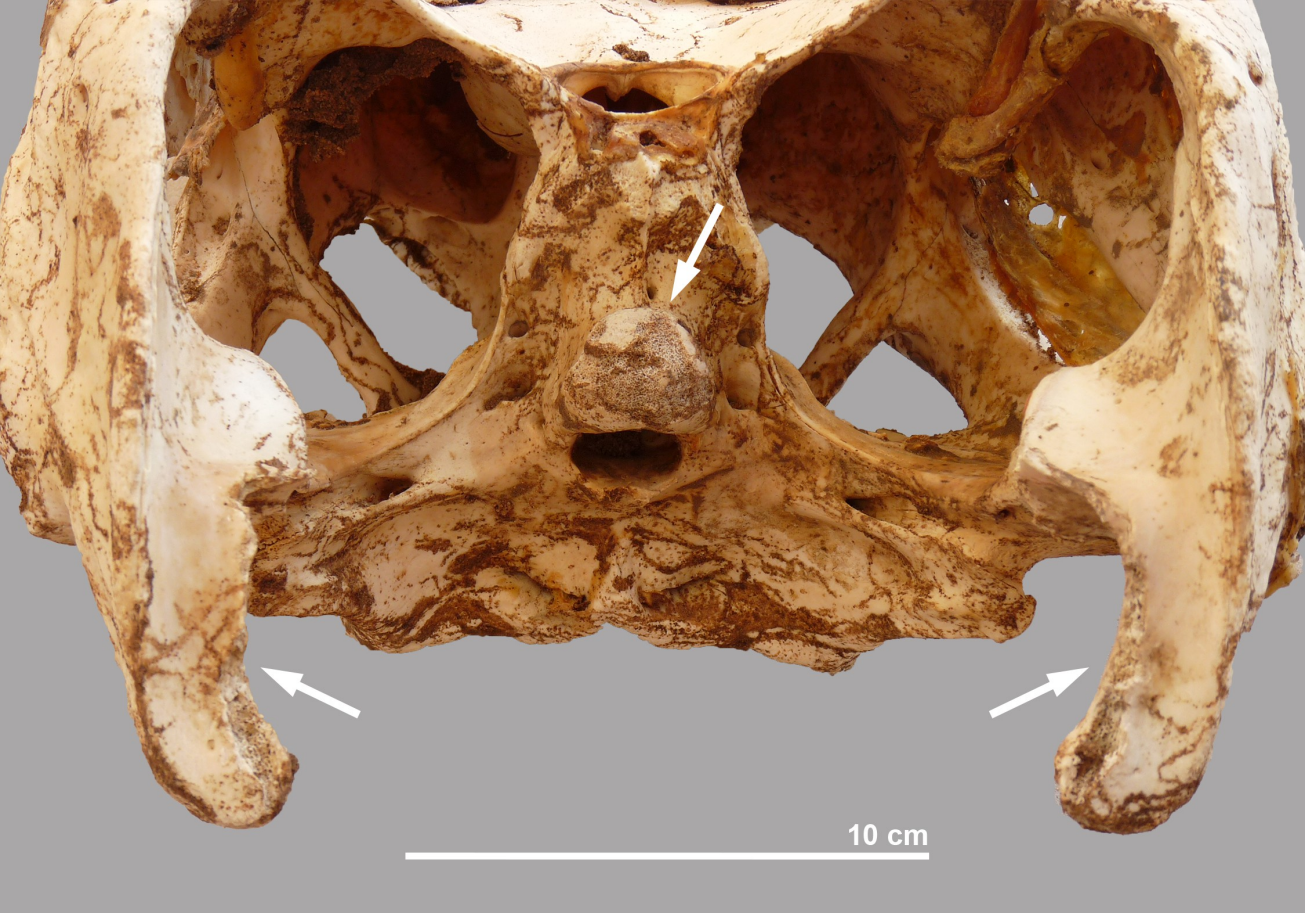
Nuchal view of the skull of crocodile #10, showing the damage to the occipital condyle and to the angulars of the lower jaws.

This is the largest isolated head found at the site and belonged to an individual with an estimated total length of 3.33 m. Remains of the skin can be observed in several areas of the lower jaws and the skull, especially on the dorsal side (Fig 15). At the snout, the nostrils are well preserved and in the posterior part of the skull, soft tissue covers the supratemporal fenestrae. The articular surface of the occipital condyle is damaged, although no clear cut mark is seen, suggesting that the bone was broken off during detachment of the head from the vertebral column. In the lower jaws, similar damage is seen on the retroarticular process of both right and left angulars (Fig 16).

#### Crocodile #3

Despite the damage to the hind skull of crocodile #3, all the defined distances could be measured. This head is from an animal with an estimated total length of 2.84 m, exactly the same size as the complete crocodile #1. The lower jaws are detached from the skull but intact. Remains of soft tissue are present and preserved in the area of the nostrils and the right eyelid (Fig 15). No marks were observed on the occipital condyle, the angulars or other bones.

#### Crocodile #6

From this specimen only the skull is present; the lower jaws are absent. The estimated total length of this individual is 2.73 m. No traces of skin are visible (Fig 15). No damage is observed to the occipital condyle or other parts of the hind skull. Unlike the other isolated skulls, all teeth are missing.

#### Crocodile #8

**Fig 17:**
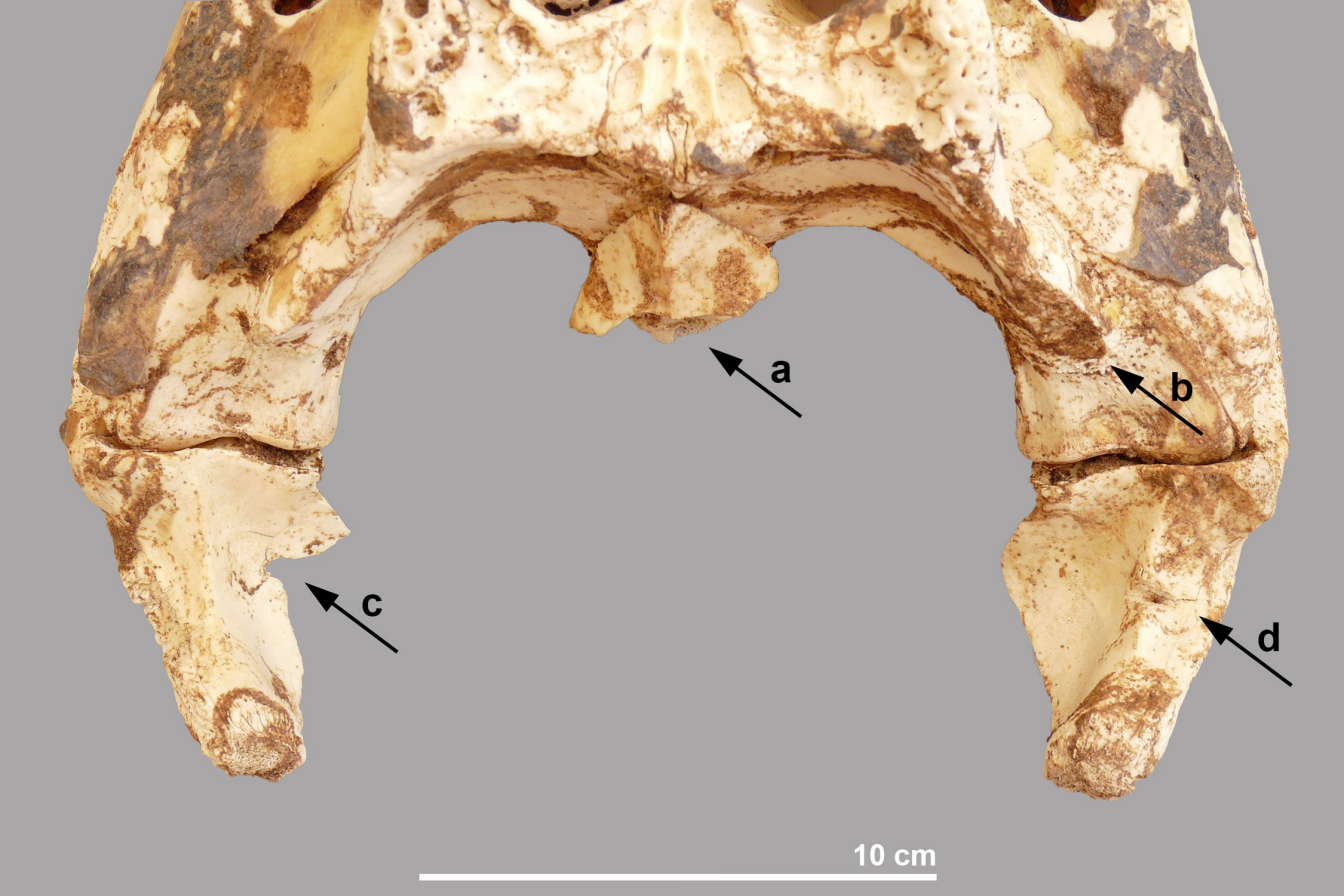
Hind part of the skull and lower jaws of crocodile #8. a: damaged occipital condyle; b: chop mark on right squamosum; c: damaged interior side of left retroarticular; d: chop mark on right articular.

This almost intact skull is from an individual with an estimated total length of 2.69 m. Skin remains are preserved over large parts of the skull and lower jaws, and in particular near the nostrils, the eye sockets, and the lateral temporal and supratemporal fenestrae (Fig 15). Of all the crocodiles found at the site, this specimen shows the most signs of processing (Fig 17). The proatlas is still attached to the occipital condyle whose articular surface was completely damaged, albeit lacking cut marks. The retroarticular process of the left lower jaw is damaged at its interior side. In the right lower jaw, a chop mark is seen on the angular, close to the distal part of the skull’s quadrate. In nuchal view, a chop mark can also be seen on the hind skull at the level of the right squamosum that is “in line” with the chop mark on the angular and thus appears to result from the same “movement” performed to separate the head from the rest of the body.

#### Crocodile #9

With its reconstructed total length of 1.85 m this is the smallest crocodile found in the assemblage. Although fully preserved, the skull of this crocodile is slightly weathered and there is no skin left at its surface (Fig 15). The occipital condyle of the skull is completely broken off; no damage can be seen in the lower jaws.

### Associated finds

**Fig 18:**
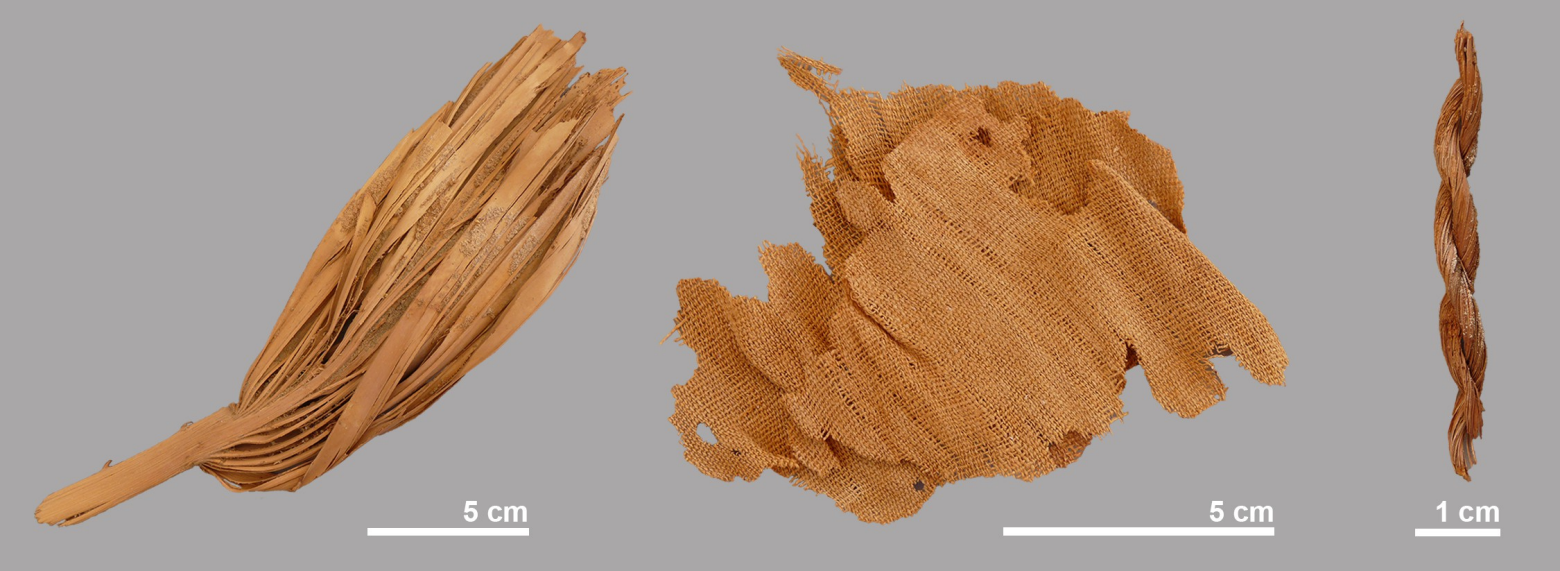
Palm leaves (left) and linen fragments (middle) associated with crocodile #5. Piece of rope (right) found near crocodile #4.

Associated to crocodiles #4, #5 and #6, leaves of palm trees (*Hyphaene* cf. *thebaica*; doum palm) were found that appear to represent material in which the animals were wrapped (Fig 18). Even scarcer are the remains of linen of which only fragments were found in association with crocodiles #5 and #11. Pieces of rope, which may have been used to hold the linen or matting together, were found with crocodile #4 and #11.

**Fig 19:**
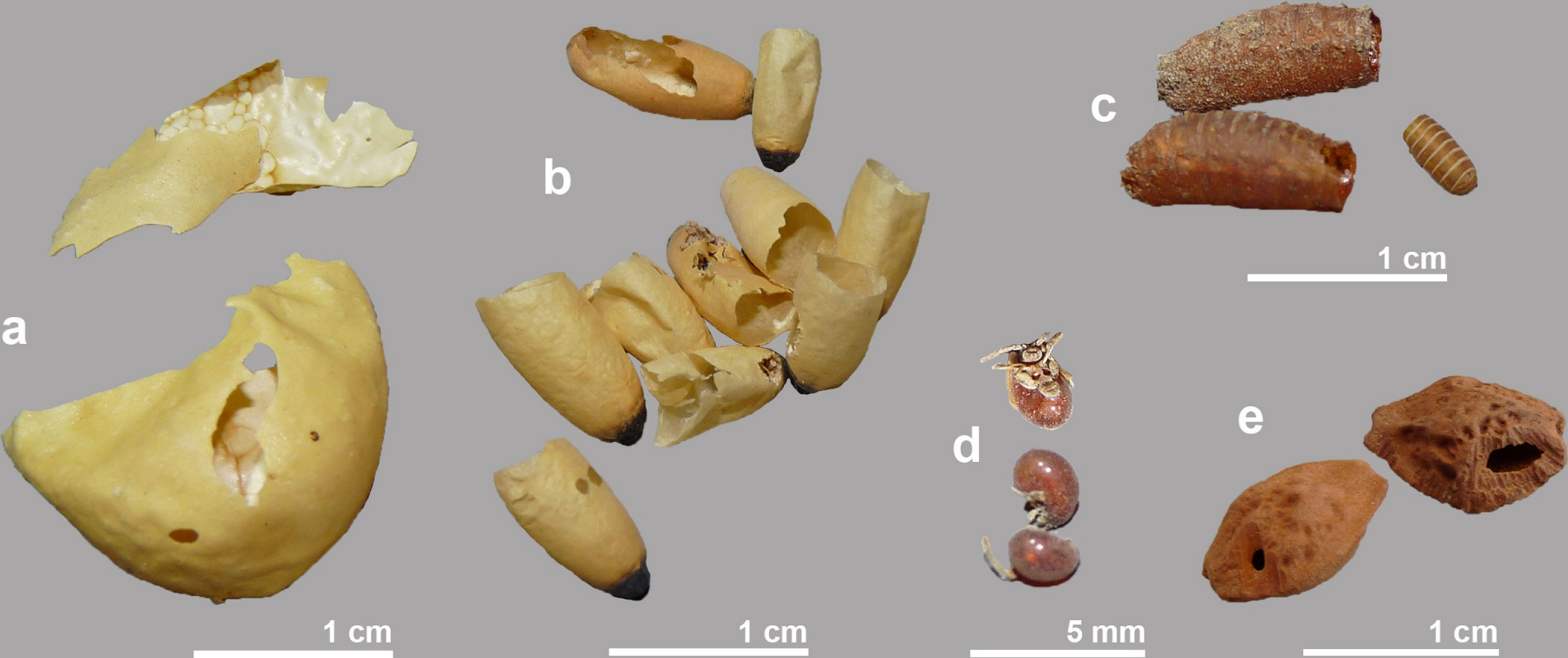
Intrusive material found associated with crocodile #5: Egg of small lizard or snake (a), pupae from ants (b), exuviae from (?) dermestids (c) and (?) oribatid mites (d). Associated with crocodile #4: two seeds of *Cordia myxa* with rodent gnaw marks (e).

Several objects could be recovered from the body cavity of crocodile #5, in addition to the gastroliths already mentioned (Fig 19). Remains of egg shells, yellowish in colour, were found (Fig 19A) corresponding to at least five eggs similar in shape and size to those discovered in one of the scanned mummies from the Musée des Confluences (MHNL 90001591) and believed to be from small lizards or snakes eaten by the crocodile [17]. Around 20 insect pupae of relatively large size, more than 1 cm in length and 4–5 mm wide, were recovered (Fig 19B). They had a very thin, fragile wall and one end, which was dark in colour, was heavier than the rest of the theca. These pupae are from ants, but we cannot give a more precise identification. Three other exuviae, belonging to two different taxa, were also found and tentatively identified as originating from dermestid beetles (Fig 19C). In addition to these insects, three specimens of mites (Fig 19D) were found reminiscent of Oribatida, a group of mites that typically live in litter and on plants. Arachnids form an important part of the diet of juvenile crocodiles, but their contribution decreases as the animals grow larger [28]. It is therefore unlikely that the oribatid mites constitute the stomach content of the crocodile. Similarly, the insects should not be seen as food of the crocodile, but as animals that invaded the crocodile when it was already a mummy. Both dermestids and ants colonize corpses relatively late, usually when the body is already desiccated [29, 30].

Some macrobotanical remains were also found in crocodile #5. There were two seeds of sedges (Cyperaceae, cf. *Cyperus*), one of pondweed (*Potamogeton* sp.) and one seed of Assyrian plum (*Cordia myxa*). A date stone (*Phoenix dactylifera*) and a seed of Assyrian plum were found in association with crocodile #1. Crocodile #4 also contained a date stone and two seeds of Assyrian plum. What is interesting about these latter seeds of Assyrian plum is that they show gnaw marks of small rodents (Fig 19E). This suggests that these seeds, and perhaps the other macrobotanical remains, are recent intrusives introduced into the body cavity by small rodents. In retrospect, one may also wonder if the reptile eggs also came from an animal that used the crocodile’s body cavity as a living space.

## Discussion

The assemblage consists of five more or less complete crocodiles with an average total length of 2.97 m (minimum 2.34 m, maximum 3.53 m). The average total length of the crocodiles from which only the skulls were deposited is 2.69 m (minimum 1.85 m, maximum 3.32 m). Below we discuss the state of preservation and completeness of these specimens, the possible cause of death of the crocodiles, the preparation techniques that may have been used, the possible provenance and methods used to capture the animals. Finally, the dating of the crocodile deposit and the species identification of the animals are discussed.

Although, as mentioned in the introduction, several hundred crocodile mummies are available for study in museums worldwide [9] not many specimens have been subjected to detailed investigation. This is undoubtedly due to the fact that observations of these mummies are complicated by the bandages and because large amounts of resin or bitumen are often applied to the animal bodies. Non-invasive imaging methods (CT-scanning and radiographing) have therefore been used on a number of mummy bundles. These imaging techniques were largely applied to verify the content of the bundles, and it turns out that not only single complete animals–corresponding to the species that was expected on the basis of the shape of the bundles–but also partial specimens, as well as multiple juvenile individuals, completely different species or even non-skeletal material occur [15]. The vast majority of crocodile mummies in museum collections are juveniles with a significant proportion of hatchlings [9, 12]. However, a few large individuals have received detailed attention and provided information relevant as a comparison with our new findings. Explicit mention of the cause of death was made in the case of three crocodiles from the Musée des Confluences [17, 18], one specimen in the Manchester Museum [15] and one in the British Museum [16]. Observations on the preparation of crocodile mummies, regarding the use of bitumen and resin, the evisceration and even the removal of other tissue are available from animals kept at the Cairo Museum [5], the Musée des Confluences [12, 17, 18] and the British Museum [16]. Radiocarbon dates are rare, and were made based on bandages in the case of specimens from the Musée des Confluences [31]. A large crocodile from Kom Ombo, kept in the British Museum [16], and two hatchlings of unknown provenance were dated based on skin fragments [32]. However, these latter dates should be treated with caution because of the predominantly piscivorous diet of crocodiles, which creates a freshwater reservoir effect. Radiocarbon dates of aquatic animals have been shown to lead to anomalously old ages with differences of up to 2000 ^14^C years [33].

In addition to these datings and the morphological observations mentioned above, genetic analysis was also carried out on a number of crocodile mummies. The crocodiles from the Egyptian Nile have usually been identified as *C*. *niloticus*, although a second species is also reported to have occurred. This additional species was first mentioned by the classical author Herodotus, who wrote that it was a smaller crocodile particularly favoured by the ancient Egyptians in their rituals because of its more docile nature. Building on this, Etienne Geoffroy Saint-Hilaire, a French naturalist, described a new species *C*. *suchus* Geoffroy 1807 that was long considered a subspecies of *C*. *niloticus*, until genetic studies showed it to be a valid species [34]. Eight mummified crocodile hatchlings from Thebes and the es-Samun caves (al-Maabda) were all assigned to *C*. *suchus* in the latter study, and subsequent aDNA analysis of an undated museum specimen about 2.5 m long, probably from Kom Ombo, also confirmed the presence of this second species in the Egyptian Nile [35]. The current distribution of *C*. *suchus* is restricted to West and Central Africa [36], showing that the species must have become extinct from Egypt. Additional genomic results from four crocodile hatchlings, without exact provenance, showed that *C*. *niloticus* was also used as a votive animal, implying that *C*. *suchus* was not the only species selected for the rituals [32].

### Completeness of the specimens

The analysis of the faunal remains showed that the completeness of the crocodiles and of the isolated skulls varied considerably. Crocodile #5, the specimen with the best preservation of soft tissue, was quite complete. There was damage to the front and hind feet and the end of the tail was also missing, showing that these are the most vulnerable body parts. This is confirmed by the representation of the skeletal elements of the four skeletons, with the foot bones and tail vertebrae missing to varying degrees (Fig 11). The absence or underrepresentation of these elements cannot be explained by the excavation techniques. The material was collected by hand, but the sandy environment made it relatively easy to retrieve the bones without sieving. Moreover, some of the missing bones are large, for example the long bones of the front and hind legs, as in crocodile #1, of which a humerus, a radius, an ulna and both femurs are missing. Nor can the absence of numerous metatarsals, whose greatest lengths vary between about 7 and 12 cm, be solely due to sampling bias. Differential preservation is also a highly unlikely explanation as the missing bones are relatively large and sturdy. So it seems that bone loss may be due to events that took place before the crocodiles were deposited.

A closer look at the skeletal representation of the crocodiles shows another pattern. The skeletons of crocodiles #11 and in particular #4 are quite complete with more or less the same elements missing as in crocodile #5, namely foot elements and terminal caudal vertebrae. In the two other crocodiles, namely crocodiles #1 and #7, it is noticeable that the vertebral columns are well represented, with the exception of the anterior cervicals and the posterior caudals. In crocodile #7, only some dorsal ribs are preserved, while all other ribs, dorsal and ventral, are totally missing. Both specimens lack the cervical ribs and the chevrons, although the vertebrae to which they were attached are present. All this indicates that we are dealing with desiccated skeletons whose axial part was still present. Probably the vertebrae were connected with desiccated tendons and intervertebral discs. The dorsal and ventral ribs, as well as the cervical ribs and chevrons had fallen off. The incompleteness of the legs is also striking in these two individuals. Apart from an isolated metacarpal, no foot bones were found in crocodiles #1 and #7. In crocodile #7 all leg bones are present (left and right radius, ulna, tibia and fibula), while in crocodile #1 the humeri and femurs are the only leg elements preserved. All these observations suggest that the overall preservation condition of crocodiles #4 and #11 may have been similar to that of crocodile #5, except that their soft tissues disintegrated. This must have happened after the crocodiles were deposited in the tomb, as their skeletal elements were still largely in articulating position and the scutes are still in place. Crocodiles #1 and #7, however, must have been low-quality mummies that were already badly damaged when they were brought into the tomb. These specimens in particular appear to have suffered from inadequate production methods.

Variable preservation conditions can also be seen in the skulls, indicating differences in the specimen preparation and handling over time. The lower jaws are still firmly attached to the cranium in crocodiles #4, #8, #9, #10 and #11 due to dried soft tissue holding them together. In the well preserved skulls of crocodiles #1 and #3, the cranium and lower jaws can be detached from each other. In all the individuals just mentioned, most of the teeth are still in place, unlike the skulls of crocodiles #6 and #7 which are almost or completely toothless. Crocodile #6 has no lower jaws and the general preservation of crocodile #7 is also poor. The cranium of the latter specimen is fractured and incomplete, and it is the only crocodile whose skull length measurements could not be taken.

### Cause of death

In all ten individuals, the outer surface of the hind skull could be observed, the area where some crocodiles mentioned in the literature show damage associated with killing. Synchroton CT-scanning recently performed on well preserved crocodile mummies, still wrapped in linen bandages and impregnated with resin, showed that the hind skull was fractured at the level of the braincase. In the case of a mummy from Kom Ombo, kept at the Musée des Confluences in Lyon (Inventory # MHNL 90001591), the size and orientation of the fractures suggest that a single blow with a stick may have caused the death of the animal that was slightly taller than 1.12 m (the tail end is missing as in our crocodile #5) [17]. A similar fracture was noted in at least two other heads from Kom Ombo, also kept at the Musée des Confluences. The trauma-induced depression was filled with resin in all three cases, indicating that the modifications took place before the mummification process and that they are therefore no post-depositional artefact. The skull of another crocodile (AEABB90, *Ancient Egyptian Animal Bio Bank*, accessed April 13^th^ 2022, https://www.mummies.manchester.ac.uk/items/show/116) of unknown provenance, measuring about 1.6 m, kept in the Manchester Museum (Museum Acquisition Number: 1772) also shows a depressed fracture [15]. Other possible indications of the cause of death, namely apparently fractured or displaced cervical vertebrae, have been reported in the literature, but should be treated with great caution as these can also occur post mortem. A natural death due to old age has been suggested for a crocodile about 4 m long with mummified crocodile hatchlings on its back kept at the British Museum (register # EA 38562) [16]. This crocodile, found at Kom Ombo, is believed to be a sacred animal rather than a votive or sacrificial offering.

The roof of all the hind skulls of the Qubbat al-Hawā crocodiles is intact, and no marks could be found that can be associated with the killing of the animals. The few chop marks and bone damage observed on three of the five isolated skulls all seem to be related to decapitation of already desiccated animals, and not to cutting off the necks of living or freshly killed animals. Among the various methods mentioned for killing crocodiles [37] the ones that do not leave clear traces and that seem to have been practicable in the past, are drowning, suffocation and heating the animals by exposing them for a long time to the sun.

**Fig 20:**
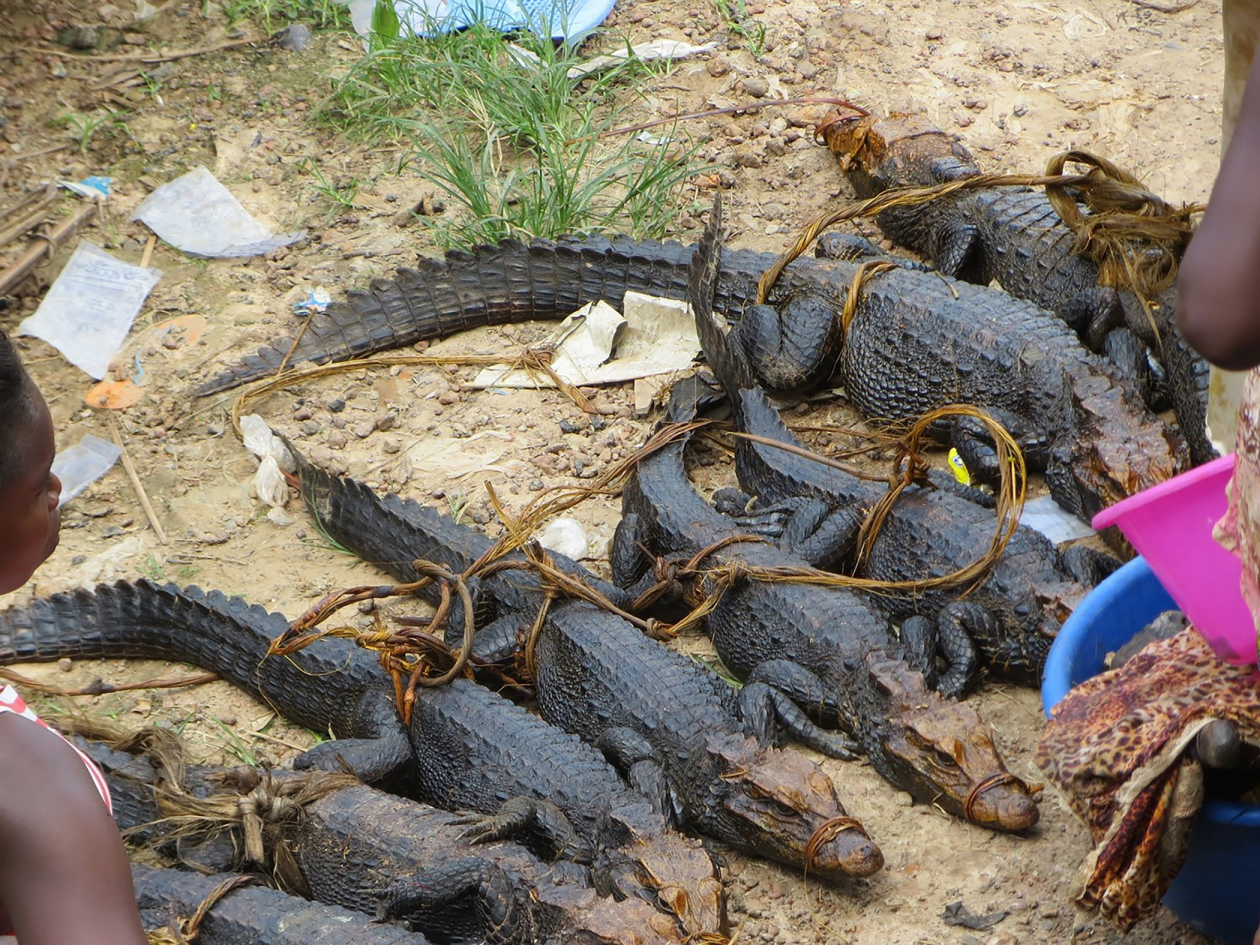
Crocodiles (*Osteolaemus osborni*) for sale at the market in Mbandaka, RD Congo, with ropes around their waists and snouts. (http://congomasquerade.blogspot.com/2019/05/urban-bushmeat-project.html; accessed 4 June 2022; © Théodore Trefon).

The above pathologies observed on the crocodile remains from Qubbat al-Hawā include a fibula with a healed fracture and the associated tibia with a thickening of the periost in crocodile #11. A similar healed fracture on a fibula of a young hippo was found at the elite cemetery HK6 of predynastic Hierakonpolis [38, 39]. It was seen as evidence that the animal had a rope on its hind leg and that the hippo broke its own leg when it tried to escape. Moreover, the fact that the fracture was healed indicates that the animal was kept in captivity for several weeks after the accident. It is highly unlikely that the healed fracture in crocodile #11 can be explained by a similar scenario. Because of the different anatomy of reptiles and mammals, it seems difficult to restrain a crocodile by attaching a rope to its hindleg. Today, when ropes are used to immobilise crocodiles, they are attached around the body, often around the waist (Fig 20). The observed pathology on the hind leg is probably the result of an injury during a fight between animals. All other pathologies on the feet and the tail of this individual, and also in crocodile #7, probably have the same cause. Trauma is highly prevalent in recent wild crocodiles, averaging 20% for all age classes combined, but rising to more than 40% in animals 3 to 4 m long [40]. The scars, wounds, fractures and even amputations observed are mainly due to territorial fights between males during the breeding season and to cannibalistic behaviour. The lesions on the pubic bones of crocodiles #4 and #11, and the healed fractures of the ventral ribs noticed in crocodile #4 may have resulted from similar violence or may also have been caused during mating. Copulation has been rarely observed in the field [40]. It has been reported in ancient Egyptian iconography in the Old Kingdom tombs of Kagemni [12] and Mehu in Saqqara [41], although the scenes may in fact represent fighting and not mating [42].

### Provenance and capture of the crocodiles

How and where the crocodiles were obtained is not easy to determine. Although crocodiles have occasionally been observed lying among rocks in Upper Egypt, they prefer river sections with slow currents and numerous sandbanks [43]. It seems unlikely that they were abundant near the site, as the Aswan area does not provide suitable living conditions for crocodiles. Since the river is very narrow, with almost no floodplain, there are very few shallow areas and riverbanks with sandy bottoms that could provide suitable basking and breeding areas.

The lack of shallow water habitats in ancient times is well illustrated by the fish remains found in the excavations on Elephantine island [44] and Syene [45]. Shallow water fishes such as tilapia and clariid catfish, commonly found on floodplains, are very rare, while taxa such as the Nile perch (*Lates niloticus*) and *Synodontis* and *Bagrus* catfish, which occur in open, deeper waters [46], are abundant. The low number of crocodile bones among the food refuse from sites on Elephantine and in Syene, ancient Aswan, has been seen as an indication of the low densities of this species [45], although the overall low contribution of hunting to the diet and food preferences may be an alternative or additional explanation. Moreover, crocodiles may have been hunted in more downstream areas of the river where the animals from Qubbat al-Hawā may also have been caught. A nearby place where crocodiles may have found suitable habitats is the area of Naq’ el-Hamdulab, on the westbank of the Nile about 5 km north of Qubbat al-Hawā. The present landscape, with its floodplain several hundred metres wide, has remained virtually unchanged since predynastic times [47].

The actual capture of crocodiles in ancient times is occasionally depicted, showing mainly netting and sometimes spearing from a boat (for a compilation see [12]). Predynastic iconography also mainly shows the capture of crocodiles with nets while spearing is usually associated with the hunting of hippos [48]. Ethnographic observations across Africa also mention these methods and, in addition, report capture in traps [49]. In many regions, including the Egyptian Nile, inhabitants of riverbanks or lake margins built defensive structures such as thorn fences or palisades to protect their livestock, but these semi-circular constructions allowed crocodiles to be caught in them at the same time. One could also use baited hooks, and a famous account by Herodotus (II, 70) mentions a technique in which a partial carcass of a pig was attached to a large hook [50]. The fisherman, standing on the shore, would then beat a restrained live pig to make it scream to attract crocodiles.

### Preparation of the crocodile mummies

There is an extensive literature on the different steps of the preparation of Egyptian human and animal mummies, for example [5, 11], and the main emphasis is on the methods involving embalming agents such as natron and resins, and the wrapping of the individuals. However, it should be remembered that the environmental conditions of Egypt are ideal for more natural mummification, namely by burying the bodies in sand pits. Forensic studies have shown that desiccation in such an environment can take place within weeks [51].

Previous observations of crocodile mummies show a wide variation in preparation methods. Synchroton CT-scans performed on a relative small (1.2 m) Roman period crocodile from Kom Ombo, kept at the Musée des Confluences, showed that all internal organs were still present [17]. This individual was wrapped in several layers of linen impregnated with a large amount of resin, which may have been a way of preventing decay. Evisceration via a cut in the abdomen has been observed in two sacred crocodiles from the Cairo Museum collection [5]. In these specimens and in other crocodiles from Kom Ombo, a large amount of resin was poured into the body, almost doubling the weight of the animals and obliterating the incision. Some specimens in the Musée des Confluences also show a ventral incision, and it appears that black resin has been applied to all animals [12]. CT-scans of the 4 metre long crocodile in the British Museum showed that this animal, from Kom Ombo, had been partially eviscerated [16]. Only the organs of the upper torso had been removed and the cavity was filled with textile. The organs of the abdomen were still in place and it was possible to observe the stomach contents with the last meal, mixed with gastroliths. On the other side of the variation in mummy preparation is the undated Kom Ombo specimen that was analysed using synchrotron microtomography [18]. In this animal, not only was complete evisceration observed, but most of the muscles and skeleton appear to have been removed. Only the foot extremities, skull and skin with its osteoderms remain.

None of the crocodiles found at Qubbat al-Hawā showed evidence of the use of resin or bitumen, and in only a few cases were small pieces of linen found (crocodile #5 and #11). In connection with crocodiles #4, #5 and #6, palm leaves were found in which the animals may once have been wrapped. Ropes that may have been used to hold this vegetal matter together were found on crocodile #4 and #11. Both the linen and palm leaves seem to have been applied when the animals were already desiccated, the question is how the individuals were dried. It is very likely that the crocodiles were desiccated by ‘deliberate natural mummification’ (sensu [52]). It is clear from the above description of the finds that the state of preservation of the crocodiles found at Qubbat al-Hawā is variable: the bone surface of the skull of the smallest individual (crocodile #9) was whitish and slightly weathered, crocodile #7 was darker in colour and had no soft tissue preserved, while all the other crocodiles showed much better preservation with skin and other soft tissue still somewhat present. All these different degrees of preservation and weathering indicate that the crocodiles did not all receive the same treatment during their preparation as mummies. We hypothesise that the observed variation is an indication that the animals were buried at different depths, during different periods of time or in different soils. This would have led to different surface exposure of the crocodile body and thus different subaerial weathering. Only in crocodile #5 were observations of the soft tissues possible and no cut marks or other indications of an evisceration were noticed. Gastroliths were still present showing that at least part of the intestines was still in situ. In the crocodile skeletons whose gastralia were preserved, no evidence of evisceration was seen either: all the gastralia were intact and no cutmarks were observed. The varying completeness of the animals, i.e. the absence of ribs, tail ends and the poor representation of limbs in some crocodiles suggest that the specimens were fragile and that they fell apart or were damaged when they were taken away from the drying site. Additional damage may also have occurred during the subsequent preparation as a mummy and later during transport to Qubbat al-Hawā, where the animals were laid out.

As mentioned above, the heads were also of varying quality: some were well preserved and in others the teeth had fallen out or the skull was fractured. Traces related to the decapitation were observed in three of the five isolated skulls. The chop marks on the cranium at the level of the squamosum and on the articular (crocodile #8) and the damage to the retroarticular processes of the angulars (crocodiles #8 and #10) indicate that a heavy tool was used in chopping off the head. In crocodiles #8, #9 and #10, the surface of the occipital condyle was damaged. In all three cases, the altered part is irregular indicating that no cutting tool but brute hand force was used to detach this part of the hind skull from the vertebral column. This seems to have happened in the final stage of decapitation after the tool was used.

Finally, it is worth recalling the way the fore and hind limbs are folded in crocodile #5. In the animals examined via CT-scanning, and also in crocodiles kept in museums whose leg positions can be observed without such analyses, it appears that both the fore and hind limbs are folded backwards. In crocodile #5 from Qubbat al-Hawā, the hind legs are folded forward under the body, and the fore leg, at least of the left limb, is pointed outwards. During the excavation, it was noted that the hind legs of crocodile #4 were also folded forward.

### Dating of the crocodiles

As mentioned above, based on their stratigraphical position, the crocodile remains can be placed between the New Kingdom and before the Byzantine period. Until radiocarbon dates are available, only circumstantial evidence can be used to speculate on a more accurate chronological attribution. As for the use and human habitation of Qubbat al-Hawā, it can be mentioned that the site served mainly as a burial place for humans during the Middle Kingdom, with reuse in the New Kingdom and Late Period. In fact, there is a hiatus in the site’s use until Byzantine times, with no evidence for human presence in the Ptolemaic and Roman periods.

Tomb QH34ll is part of a small group of graves constructed during the late First Intermediate Period/early Middle Kingdom (QH33bb [53]; QH34cc [54]; QH34ff, QH34gg, QH34hh, QH34ii, QH34jj and QH34kk [25]). Some were reoccupied during the first half of the 18^th^ Dynasty, but most were reused for multiple burials during the 6^th^-5^th^ centuries BC. This use coincides with neighbouring reuse of the monumental tombs QH31 and QH33 [55]. After the 5^th^ century, Qubbet al-Hawā appears to have been abandoned as a burial ground in favour of the Wadi Samman, where an Egyptian-Italian mission has been conducting excavations since 2018 [56]. Preliminary results of this archaeological project has shown that the funerary use of this site runs from the Ptolemaic period (3^rd^ century BC) till the 1^st^/2^nd^ century AD [57]. It is precisely from this later period that numerous crocodile mummies have been reported from Kom Ombo (50 km north of Aswan) and Esna (about 150 km), the localities closest to Qubbat al-Hawā where crocodile cemeteries have been reported. The crocodile mummies at the Musée des Confluences, dated with radiocarbon [17, 31] yielded mainly late dates, both for Kom Ombo and Esna as well as for a series of crocodiles of unknown provenance. The calibrated dates (2σ) for three crocodiles from Kom Ombo range between the 1^st^ and the mid-3^rd^ century AD, the specimen from Esna is dated to 52 BC-71 AD, and all but one of the four unprovenanced crocodiles fall in the same range. A head of a crocodile without provenance was dated to 2255 ± 30 BP (or 396 cal BC (35.1%) 348 cal BC; 316 cal BC (60.3%) 208 cal BC; 2σ). The even older dating (2518 ± 27 BP; 792‑542 cal BC; 2σ) obtained on the skin of the 4 m long crocodile mummy from Kom Ombo, preserved at the British Museum [16], is unreliable due to the freshwater reservoir effect. Radiocarbon dating of two hatchlings of unknown provenance from the Yale Peabody Museum of Natural History was also done on skin [32]. The calibrated (2σ) dates 169 BC-8 AD and 54 BC-82 AD should also be considered too old.

Of importance for the chronological attribution of the crocodiles from Qubbat al-Hawā is the absence of any trace of resin, a substance that was abundantly applied to all crocodile mummies from Kom Ombo and Esna [12]. In the diachronic series of baboon and ibis mummies from Tuna el-Gebel, there is clear evidence of a shift in embalming techniques. In Saitic times (664–525 BC), baboons were prepared by burying them in sandy sediments, followed by unearthing and removing the remaining soft tissues, and finally wrapping them in linen cloth [58]. The same technique was used in Persian times (525–332 BC), but from then on small amounts of resin (turpentine and/or bitumen) were applied. However, it was only during the reign of Ptolemy I (304–284 BC) that larger amounts of resin were used, eliminating the need to start mummy production with maceration or burial in sand. The large series of ibis mummies also show that before the 30^th^ Dynasty (380–343 BC), bitumen or resin was rarely used [59, 60]. The method of preparation of the crocodile mummies from Qubbat al-Hawā suggests a pre-Ptolemaic dating for the deposit, a hypothesis consistent with the final phase of funerary use of Qubbet al-Hawā in 5^th^ century BC (see above).

### Species identification

As ancient DNA studies could not be performed on the specimens from Qubbat al-Hawā, we examined the morphological criteria that were described by Geoffroy Saint-Hilaire [61, 62] as diagnostic for *C*. *suchus*. The distinguishing criteria he defined were based on the examination and comparison of a single specimen of a *C*. *niloticus* measuring 1.28 m and of a *C*. *suchus* measuring 1.19 m. The two main criteria that can be applied to the material from Qubbat al-Hawā are the skull shape and the number and arrangement of the dermal scutes. Since both specimens used by Geoffroy Saint-Hilaire are the same length, the observed differences cannot be size-related. It is also doubtful whether the different skull proportions observed in the two specimens are due to sexual dimorphism. A study of sexual dimorphism and allometric growth in a south American crocodile showed that morphometric differences do not occur in animals smaller than 2 metres in length [63]. Crocodile #5 is the only specimen in which the dermal scutes can be observed within the skin (Fig 6). Behind the four post-occipital scutes (called ‘plaques nuchales’ [62]), six nuchal scutes (called ‘plaques cervicales’ [62]) are arranged in two rows. The anterior row consists of four larger scutes, while the posterior row consists of two smaller scutes. This arrangement is typical of *C*. *niloticus*; in *C*. *suchus* there are eight nuchal scutes in two rows [62].

**Fig 21:**
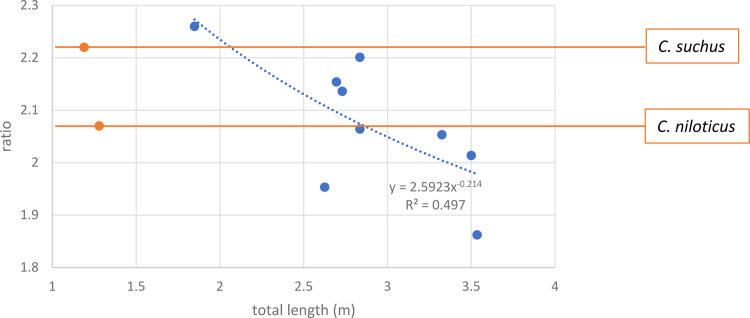
Ratio of the lateral length and the posterior width of the skull versus the estimated total length of the crocodiles from Qubbat al-Hawā. The trend line shown is the power curve for all specimens from the site. The specimens of *C*. *suchus* and *C*. *niloticus* described by Geoffroy Saint-Hilaire are coloured orange.

The two species also differ in skull shape, with that of *C*. *suchus* being more elongated. Geoffroy Saint-Hilaire [62] mentions that the ratio of the greatest lateral length of the skull to the greatest posterior breadth, measured across the quadratojugale, is 2.07 in *C*. *niloticus* and 2.22 in *C*. *suchus*. These ratios could be established for all individuals found in Qubbat al-Hawā, except for crocodile #7 with its fractured, incomplete skull. The ratio greatest lateral length to greatest posterior breadth of the skulls varies from 1.86 for a 3.54 metre animal to 2.26 for a 1.85 metre crocodile (Table 2). When the ratios are plotted against the total length of the crocodiles, although the coefficient of determination is low, it appears that their values decrease as the animals grow larger (Fig 21). This means that the heads of crocodiles become proportionally wider as their overall length increases. In any case, the two specimens examined by Geoffroy Saint-Hilaire [62] were of similar length and the ratios greatest lateral length to greatest posterior breadth are clearly different. The specimen identified by Geoffroy Saint-Hilaire as *C*. *suchus* plots relatively close to the smallest individual from Qubbet al-Hawā. It is tempting to interpret all but the two specimens with the lowest ratio (1.95 and 1.86, in the lower part of the graph) as *C*. *suchus*. Indeed, the individual with the ratio 1.95 (= crocodile #5) had already identified as *C*. *niloticus* by its scutes. If the two aforementioned specimens are omitted, an equation with a much better correlation is obtained (y = 2.536x^-0.173^; R^2^ = 0.7534). The *C*. *niloticus* described by Geoffroy Saint-Hilaire and the two omitted specimens from Qubbet al-Hawā, although only three individuals, fit very well on a power curve (R^2^ = 0.9696). The data obtained seem to indicate that the two species may have been present at the site. However, this would mean–if our species designations are correct–that *C*. *suchus* could reach much larger sizes than mentioned by ancient writers and could even surpass the 2.5 m length of a *C*. *suchus* recently identified by genetic analysis.

**Table 2:** Ratio of the greatest lateral length of the skull to the greatest posterior breadth measured across the quadratojugale, and the estimated total length (TL) of each individual.

Crocodile	#11	#5	#4	#10	#1	#6	#8	#3	#9
Ratio	1.86	1.95	2.01	2.05	2.06	2.14	2.15	2.20	2.26
TL (m)	3.54	2.63	3.50	3.33	2.84	2.73	2.70	2.84	1.85

## Conclusion

The mummified crocodiles from Qubbat al-Hawā differ from mummies thus far recorded from other localities. There was no evidence of the use of bitumen or pitch, for evisceration or any other special preparation technique. It is assumed that the animals were laid on the surface or, more likely, buried in a sandy environment that allowed the bodies to dry out naturally. The use of linen and wrapping in mats was documented for some specimens, but it is unclear whether this was the case for all animals, as this organic material may have been eaten by insects. Unlike the crocodile mummies reported in the literature so far in which the fore and hindlegs are oriented backward, in the two mummies of Qubbat al-Hawā in which the orientations could be properly observed, the hindlegs were folded forward and at least one of the forelegs was oriented laterally in a more or less natural position. The most striking feature of this collection of crocodiles is the wide variation in the state of preservation of the mummies. The only individual whose skin and other soft tissue were still preserved showed damage at the end of the tail and the extremities of the feet. The same damage to those body parts is evident from the loss of skeletal elements in two fairly well-preserved skeletons. Two more crocodiles were very incomplete with loss of the anterior and posterior most parts of the vertebral column and most ribs, as well as the legs. This pattern is not related to preservation conditions at the site, but indicates that some of the dried crocodile carcasses were already of poor quality, disarticulated or decomposed, when they were deposited. Similar variation was observed in the state of preservation of the five isolated heads, with specimens whose skin was still attached and one without any teeth. The chop marks and damage pattern observed in the hind skulls show that the animals were decapitated when the crocodiles were already desiccated.

Finally it should be mentioned that no evidence was found on how the animals were killed or where they may have been caught. The crocodiles varied in length between 1.8 and 3.5 m and it seems that both *C*. *niloticus* and *C*. *suchus* are present, based on the morphological criteria described as diagnostic. It would be ideal to test this further with genomic analysis and radiocarbon dating of the animals would also be worthwhile. For now, the crocodile deposit is assumed to be pre-Ptolemaic, based on the lack of evidence of resin or bitumen use, and on the archaeological information about human presence at the site.

## Supporting information

S1 Fig Measuring distances of postcranial skeletal elements. All paired elements shown are from the left side of the body. 1) atlas, anterior view; BFcr, breadth of the Facies articularis cranialis. 2) axis, anterior view; BFcr, breadth of the Facies articularis cranialis. 3) axis, left lateral view; GL, greatest length of the corpus. 4) hyoid, dorsal view; GL, greatest length. 5) coracoid, lateral view; GL, greatest length; SD: smallest diameter. 6) scapula, lateral view; GL, greatest length; GLo, greatest oblique length; GBinf, greatest breadth inferior end; SD: smallest diameter. 7) calcaneus, dorsal view; GL, greatest length. 8) astragalus, anterior view; GL, greatest length.9) ischium, lateral view; GL, greatest length. 10) pubis, lateral view; GLo, greatest oblique length; Bp, greatest breadth proximal end; SD, smallest diameter; Bd, greatest breadth distal end. 11) ilium, lateral view; GL, greatest length.
(TIF)

S2 Fig Schematic representation of the crocodile skeleton with indication of its main elements. (TIF)

S1 Table Measurements (in mm) taken on the postcranial bones. The measuring distances are shown in S1 Fig. Values in brackets are approximate.
(XLSX)

S2 Table Absolute numbers of skeletal elements expected in a crocodile skeleton [1], and the proportion observed in the individuals #4, #11, #7 and #1. (XLSX)

## References

[1] Jiménez Serrano A, Alba Gómez J, de La Torre Robles Y, García González LM, Barba Colmenero V, Caño Dortez A, et al. Proyecto Qubbet el-Hawa: Trabajos arqueólogicos en las tumbas QH32, QH33, QH34aa, QH34bb, QH122, QH35p y QH36. Novena Campaña (2017). Boletín de la Asociación Española de Egiptología (BAEDE). 2017;26:13–110.

[2] Van Neer W. Faunal report. In: Jiménez Serrano A et al. Proyecto Qubbet el-Hawa: Trabajos arqueólogicos en las tumbas QH32, QH33, QH34aa, QH34bb, QH122, QH35p y QH36. Novena Campaña (2017). Boletín de la Asociación Española de Egiptología (BAEDE). 2017;26:80–82.

[3] Edel E. Bericht über die Arbeiten in den Gräbern der Qubbet el-Hawa bei Assuan 1961, 1962 und 1963. Annales du Service des antiquités de l’Egypte. 1968;60:77–94.

[4] Kessler D. Die heiligen Tiere und der König. Teil I: Beiträge zur Organisation, Kult und Theologie der spätzeitlichen Tierfriedhöfe. Wiesbaden, Otto Harrassowitz; 1989.

[5] Ikram S. Manufacturing divinity. The technology of mummification. In Ikram S, editor. Divine creatures: animal mummies from ancient Egypt. Cairo: The American University in Cairo Press; 2005. pp. 16–43.

[6] Bresciani E. “Sobek, Lord of the Land of the Lake”. In Ikram S, editor. Divine creatures: animal mummies from ancient Egypt. Cairo: The American University in Cairo Press; 2005. pp. 199–206.

[7] Yoyotte P. Crocodile. In: Vernus P, Yoyotte J, editors. Bestiaire des pharaons. Paris: Librairie Académique Perrin; 2005. pp. 209–240.

[8] De Meulenaere H. Sébek à Elkab. Chronique d’Egypte. 1969;44:13–21.

[9] Berruyer C. Synchrotron X-ray imaging contribution to the study of animal mummification in ancient Egypt: archaeozoological study of crocodile mummies (Ptolemaic and Roman periods). Doctoral dissertation, Université Lumière Lyon 2. 2021.

[10] Nicolotti M, Postel L. 1994. L’animal et le monde de l’au-delà: les momies du Muséum d’Histoire Naturelle de Lyon. Bulletin du Cercle lyonnais d’égyptologie Victor Loret. 1994;8:35–48.

[11] Ikram S. Animal Mummies in ancient Egypt and South America. In: Shin DH, Bianucci R, editors. The handbook of mummy studies. Singapore: Springer; 2020. pp. 629–651.

[12] Nicolotti M, Robert L. Les crocodiles momifiés du muséum de Lyon. Nouvelles archives du Muséum d’histoire naturelle de Lyon. 1994;32:4–62.

[13] Lortet L, Gaillard C. La faune momifiée de l’ancienne Égypte. Archives du Museum d’Histoire naturelle de Lyon. 1903;8. Lyon.

[14] Lortet L, Gaillard C. La faune momifiée de l’ancienne Égypte. Archives du Museum d’Histoire naturelle de Lyon. 1909;10. Lyon.

[15] McKnight LM, Atherton-Woolham S, Adams JE. Imaging of ancient Egyptian animal mummies. RadioGraphics. 2015;35:2108–2120. doi: 10.1148/rg.2015140309 26562240

[16] Anderson J, Antoine D. Scanning Sobek. Mummy of the Crocodile God. In: Porcier S, Ikram S, Pasquali S, editors, Creatures of earth, water and sky. Essays on animals in ancient Egypt and Nubia. Leiden: Sidestone Press; 2019. pp. 31–37.

[17] Porcier SM, Berruyer C, Pasquali S, Ikram S, Berthet D, Tafforeau P. Wild crocodiles hunted to make mummies in Roman Egypt: Evidence from synchrotron imaging. J Archaeol Sci. 2019;110:105009.

[18] Berruyer C, Porcier SM, Tafforeau P. Synchrotron “virtual archaeozoology” reveals how Ancient Egyptians prepared a decaying crocodile cadaver for mummification. PLoS One. 2020;15(2): e0229140. doi: 10.1371/journal.pone.0229140 32084197 PMC7034907

[19] Chudzik P, Iwaszczuk U. “A Crocodile Spirit, Crocodile-Faced”: discovery of crocodile remains in the Early Middle Kingdom tombs of the North Asasif necropolis in western Thebes (Egypt), Journal of African Archaeology. 2022;20:107–124. doi: 10.1163/21915784-bja10016

[20] De Meyer M, Van Neer W, Peeters C, Willems H. The role of animals in the funerary rites at Dayr al-Barshā. Journal of the American Research Center in Egypt. 2005;42:45–71.

[21] Peters J, von den Driesch A. Archäozoologisch-kulturhistorische Auswertung frühgeschichtlicher Krokodilknochen aus Al-M’abda (Mittelägypten). Bonn Zool Beitr. 1999;50:211–219.

[22] Boessneck J, von den Driesch A. Die Tierknochenfunde aus den Pavian- und Ibisgalerien von Tuna el-Gebel. In: Boessneck J, editor. Tuna el-Gebel I: die Tiergalerien. Hildesheimer Ägyptologische Beiträge. 1987;24:39–221. Hildesheim: Gerstenberg.

[23] Gautier A, Hendrickx S. Vultures and other animal remains from a tomb in the Elkab necropolis, Upper Egypt: an exercise in salvage archaeozoology. In: Becker C, Manhart H, Peters J, Schibler J, editors. Historia animalium ex ossibus. Beiträge zur Paläoanatomie, Archäologie, Ägyptologie, Ethnologie und Geschichte der Tiermedizin, Festschrift für Angela von den Driesch zum 65. Geburtstag. Internationale Archäologie, Studia honoraria. 1999;8:161–178. Rahden: Marie Verlag.

[24] Gautier A. Animal mummies and remains from the necropolis of Elkab (Upper Egypt). Archaeofauna. 2005;14:139–170.

[25] Jiménez Serrano A, Barba Colmenero V. Work on the exterior of Tombs QH34AA and QH34EE: The new tombs QH34FF, QH34GG, QH34II, QH34JJ, QH34KK and QH34LL. In: Jiménez Serrano A, Tooley AMJ, Alba Gómez JM, Lechuga Ibáñez C, editors. Results of the 2019 research season at Qubbat al-Hawā, Arqueologías Egiptología. 2021;3: 3–42. Editorial Universidad de Jaén.

[26] Hutton JM. Growth and feeding ecology of the Nile Crocodiles *Crocodylus niloticus* at Ngezi, Zimbabwe. J Anim Ecol. 1987;56:25–38.

[27] Fukuda Y, Saalfeld K, Lindner G, Nichols T. Estimation of total length from head length of saltwater crocodiles (*Crocodylus porosus*) in the Northern Territory, Australia. J Herpetol. 2013;47:34–40.

[28] Wallace KM, Leslie AJ. Diet of the Nile Crocodile (*Crocodylus niloticus*) in the Okavango Delta, Botswana. J Herpetol. 2008;42:361–368.

[29] Abd El-Bar MM, Sawaby RB, El-Hamouly H, Hamdy R. A preliminary identification of insect successive wave in Egypt on control and zinc phosphide-intoxicated animals in different seasons. Egypt J Forensic Sci. 2016;6: 223–234.

[30] Eubanks MD, Lin C, Tarone AM. The role of ants in vertebrate carrion decomposition. Food Webs. 2019;18: e00109. doi: 10.1016/j.foodweb.2018.e00109

[31] Richardin P, Porcier S, Ikram S, Louarn G, Berthet D. Cats, crocodiles, cattle, and more: Initial steps toward establishing a chronology of ancient Egyptian animal mummies. Radiocarbon. 2017;59:595–607.

[32] Hekkala ER, Colten R, Cunningham SW, Smith O, Ikram S. Using mitogenomes to explore the social and ecological contexts of crocodile mummification in ancient Egypt. Bull Peabody Mus Nat Hist. 2022; 63:3–14. doi: 10.3374/014.063.0101

[33] Philippsen B. The freshwater reservoir effect in radiocarbon dating. Her Sci. 2013;1:24. doi: 10.1186/2050-7445-1-24

[34] Hekkala ER, Shirley MH, Amato G, Austin JD, Charter S, Thorbjarnarson J et al. An ancient icon reveals new mysteries: mummy DNA resurrects a cryptic species within the Nile crocodile. Mol Ecol. 2011;20:4199–4215. doi: 10.1111/j.1365-294X.2011.05245.x 21906195

[35] Hekkala ER, Aardema ML, Narechania A, Amato G, Ikram S, Shirley MH, et al. The secrets of Sobek–A crocodile mummy mitogenome from ancient Egypt. J Archaeol Sci Rep. 2020;33:102483. doi: 10.1016/j.jasrep.2020.102483

[36] Cunningham SW, Shirley MH, Hekkala ER. Fine scale patterns of genetic partitioning in the rediscovered African crocodile, *Crocodylus suchus* (Saint-Hilaire 1807). PeerJ. 2016;4:e1901. doi: 10.7717/peerj.1901 27114867 PMC4841213

[37] IUCN Expert Panel. Humane euthanasia/killing methods for crocodilians. Expert Panel to assess humane methods of killing reptiles in trade. IUCN; 2013. Available from: https://www.iucncsg.org/365_docs/attachments/protarea/Euth-3e9afc67.pdf.

[38] Van Neer W, Linseele V. Animal hospital: healed animal bones from HK6. Nekhen News. 2009;21:11–12.

[39] Van Neer W, Udrescu M, Linseele V, De Cupere B, Friedman R. Traumatism in the wild animals kept and offered at predynastic Hierakonpolis, Upper Egypt. Int J Osteoarchaeol. 2017;27:86–105.

[40] Cott HB. Scientific results of an inquiry into the ecology and economic status of the Nile crocodile (*Crocodilus niloticus*) in Uganda and northern Rhodesia. Transactions Zoological Society London. 1961;29:211–356.

[41] Altenmüller H. Die Wanddartstellungen im Grab des Mehu in Saqqara. Archäologische Veröffentlichungen (Deutsches Archäologisches Institut, Abteilung Kairo). 1998;42. Mainz: von Zabern.

[42] Ikram S. Animal Mating Motifs in Tombs of the Old Kingdom. Göttinger Miszellen 1991;124:51–68.

[43] Anderson J. Zoology of Egypt: Reptiles and Batrachia. London; 1898.

[44] Katzmann L. Tierknochenfunde aus Elephantine in Oberägypten (Grabungsjahre 1976 bis 1986/87). Vögel, Reptilien, Fische und Mollusken. Doctoral dissertation, LMU München. 1990.

[45] Sigl J. Syene II—Die Tierfunde aus den Grabungen von 2000–2009. Ein Beitrag zur Umwelt- und Kulturgeschichte einer oberägyptischen Stadt von der pharanoischen Spät- bis in die Mameluckenzeit. Beiträge zur Ägyptischen Bauforschung und Altertumskunde. 2017;19. Gladbeck: PeWe-Verlag.

[46] Van Neer W. Evolution of prehistoric fishing in the Nile Valley. Journal of African Archaeology. 2004;2:251–269.

[47] Gatto M, Darnell J, De Dapper M, Gallorini C, Gerisch R, Giuliani S, et al. Archaeological investigation in the Aswan-Kom Ombo region (2007–2008). Mitteilungen des Deutschen Archäologischen Instituts Abteilung Kairo. 2009;65,9–49. Berlin/NY: de Gruyter.

[48] Hendrickx S. L’iconographie de la chasse dans le contexte social prédynastique. Archéo-Nil. 2010;20:108–136.

[49] Pooley S. A Cultural Herpetology of Nile Crocodiles in Africa. Conserv Soc. 2016;14:391–405. doi: 10.4103/0972-4923.197609

[50] Herodotus. The Histories, translated by Aubrey de Sélincourt and revised with introduction and notes by John Marincola. London: Penguin Books; 2003.

[51] Piombino-Mascali D, Carr H. Dried, tanned, frozen, embalmed, smoked: A glimpse into mummification mechanisms. In: Shin DH, Bianucci R, editors. The handbook of mummy studies. Singapore: Springer; 2020. pp. 41–58.

[52] David AR. Mummification. In: Nicholson PT, Shaw I, editors. Ancient Egyptian materials and technology. Cambridge: Cambridge University Press; 2000. pp. 372–389.

[53] Jiménez Serrano A, Martínez de Dios JL, Alba JM, Chpon L, García González L, López-Obregón Silvestre T, et al. Cuarta campaña (2012) de excavaciones en las tumbas 33 y 34 de la necrópolis de Qubbet el-Hawa (Asuán, Egipto). Boletín de la Asociación Española de Egiptología (BAEDE). 2012;21:107–136.

[54] Jiménez Serrano A, Alba Gómez JM, Ayora Cañada MJ, Barba Colmenero V, Botella López M, et al. Proyecto Qubbet el-Hawa: las tumbas n° 31, 34cc y 35p. Séptima campaña (2015). Boletín de la Asociación Española de Egiptología. 2015; 24:7–88.

[55] de la Torre Robles Y. Late Period Coffins from Qubbet el-Hawa Tomb 33. In: Strudwick H, Dawson J, editors. Ancient Egyptian coffins: past—present—future. Oxford: Oxbow Books; 2019. pp. 168–174. doi: 10.2307/j.ctvh9w0cw.22

[56] https://aswan-milano.org

[57] https://aswan-milano.org/lo-scavo/

[58] von den Driesch A, Kessler D, Peters J. Mummified baboons and other primates from the Saitic-Ptolemaic animal necropolis of Tuna el-Gebel, Middle Egypt. In: Grupe G, Peters J, editors. Conservation Policy and Current Research. Documenta Archaeobiologiae. 2004;2: 231–278. Rahden/Westf.: Leidorf.

[59] von den Driesch A, Kessler D, Steinmann F, Berteaux V, Peters J. Mummified, deified and buried at Hermopolis Magna—the sacred birds from Tuna El-Gebel, Middle Egypt. Ägypten und Levante / Egypt and the Levant. 2005;15:203–244.

[60] Kessler D, Nur el-Din AH. Tuna el-Gebel: millions of Ibises and other animals. In Ikram S, editor. Divine creatures: animal mummies from ancient Egypt. Cairo: The American University in Cairo Press; 2005. pp. 120–163.

[61] Geoffroy Saint-Hilaire E. Description de deux crocodiles qui existent dans le Nil, comparés au crocodile de Saint-Domingue. Ann Mus Hist Nat. 1807;10:67–86, 264 + 2 pl.

[62] Geoffroy Saint-Hilaire E. Description des crocodiles d’Egypte. Description de l’Égypte, ou Recueil des observations et des recherches qui ont été faites en Égypte pendant l’expédition de l’armée française, publié par les ordres de sa majesté l’empereur Napoléon le grand. Histoire naturelle. 1829;24:401–571.

[63] Barrios-Quiroz G, Casas-Andreu G, Escobedo-Galván AH. Sexual size dimorphism and allometric growth of Morelet’s crocodiles in captivity. Zoolog Sci. 2021;29:198–203. 10.2108/zsj.29.198 22379988

