# Supplementary figures and images for: Newly discovered crocodile mummies of variable quality from an undisturbed tomb at Qubbat al-Hawā (Aswan, Egypt)
Source: PLoS One. 2023 Jan 18;18(1):e0279137. doi: 10.1371/journal.pone.0279137 (PMC9848469; doi:10.1371/journal.pone.0279137)

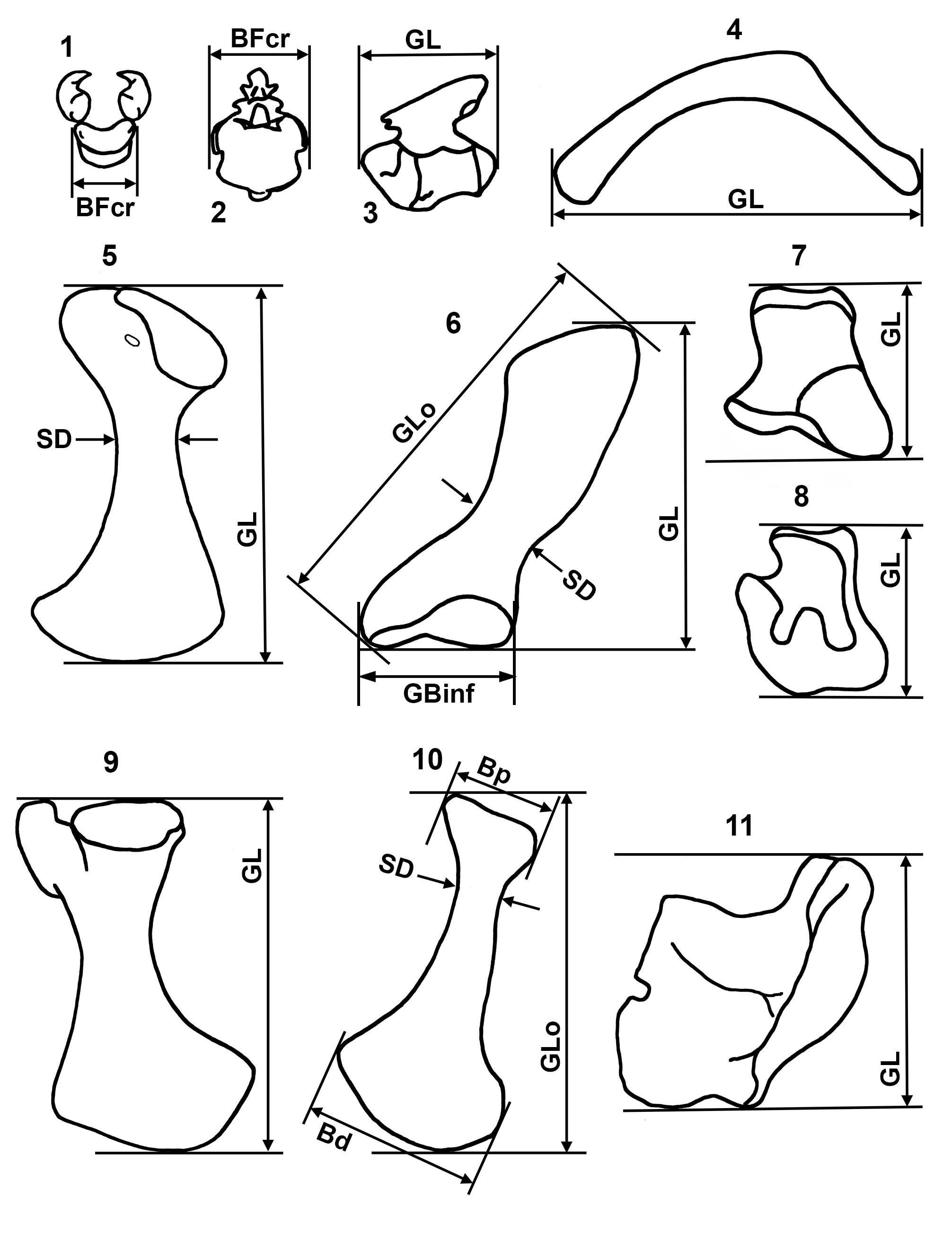

Supplement: S1 Fig — All paired elements shown are from the left side of the body. 1) atlas, anterior view; BFcr, breadth of the Facies articularis cranialis. 2) axis, anterior view; BFcr, breadth of the Facies articularis cranialis. 3) axis, left lateral view; GL, greatest length of the corpus. 4) hyoid, dorsal view; GL, greatest length. 5) coracoid, lateral view; GL, greatest length; SD: smallest diameter. 6) scapula, lateral view; GL, greatest length; GLo, greatest oblique length; GBinf, greatest breadth inferior end; SD: smallest diameter. 7) calcaneus, dorsal view; GL, greatest length. 8) astragalus, anterior view; GL, greatest length.9) ischium, lateral view; GL, greatest length. 10) pubis, lateral view; GLo, greatest oblique length; Bp, greatest breadth proximal end; SD, smallest diameter; Bd, greatest breadth distal end. 11) ilium, lateral view; GL, greatest length. (TIF) [file pone.0279137.s001.tif]

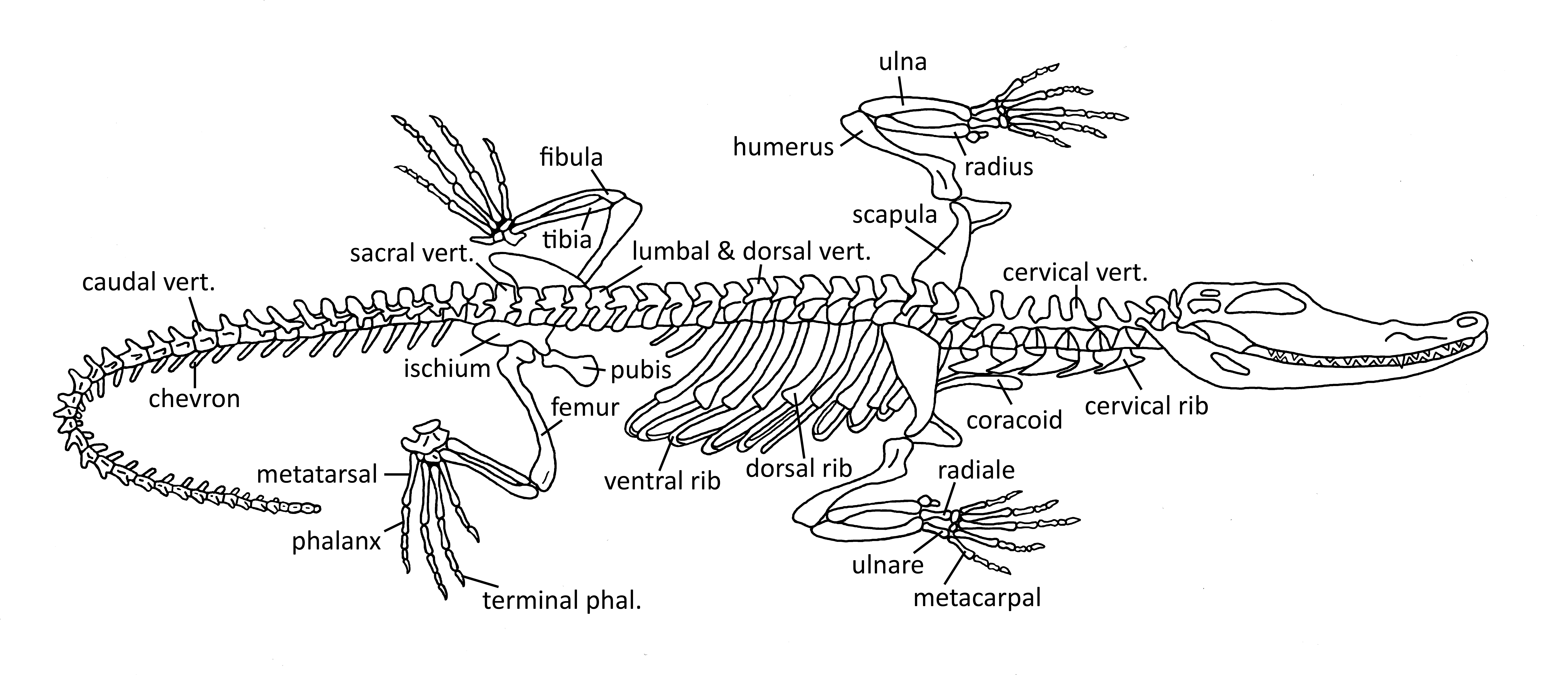

Supplement: S2 Fig — (TIF) [file pone.0279137.s002.tif]
